# scAIDE: clustering of large-scale single-cell RNA-seq data reveals putative and rare cell types

**DOI:** 10.1093/nargab/lqaa082

**Published:** 2020-10-09

**Authors:** Kaikun Xie, Yu Huang, Feng Zeng, Zehua Liu, Ting Chen

**Affiliations:** Institute for Artificial Intelligence, Department of Computer Science and Technology, Tsinghua University, Beijing 100084, China; Tsinghua-Fuzhou Institute of Digital Technology, Beijing National Research Center for Information Science and Technology, Tsinghua University, Beijing 100084, China; Institute for Artificial Intelligence, Department of Computer Science and Technology, Tsinghua University, Beijing 100084, China; Tsinghua-Fuzhou Institute of Digital Technology, Beijing National Research Center for Information Science and Technology, Tsinghua University, Beijing 100084, China; Department of Automation, Xiamen University, Xiamen 361005, China; National Institute for Data Science in Health and Medicine, Xiamen University, Xiamen 361005, China; Center for Computational and Integrative Biology, Massachusetts General Hospital, Harvard Medical School, Boston, MA 02114, USA; Department of Molecular Biology, Massachusetts General Hospital, Harvard Medical School, Boston, MA 02114, USA; Broad Institute of Massachusetts Institute of Technology and Harvard, Cambridge, MA 02142, USA; Institute for Artificial Intelligence, Department of Computer Science and Technology, Tsinghua University, Beijing 100084, China; Tsinghua-Fuzhou Institute of Digital Technology, Beijing National Research Center for Information Science and Technology, Tsinghua University, Beijing 100084, China

## Abstract

Recent advancements in both single-cell RNA-sequencing technology and computational resources facilitate the study of cell types on global populations. Up to millions of cells can now be sequenced in one experiment; thus, accurate and efficient computational methods are needed to provide clustering and post-analysis of assigning putative and rare cell types. Here, we present a novel unsupervised deep learning clustering framework that is robust and highly scalable. To overcome the high level of noise, scAIDE first incorporates an autoencoder-imputation network with a distance-preserved embedding network (AIDE) to learn a good representation of data, and then applies a random projection hashing based *k*-means algorithm to accommodate the detection of rare cell types. We analyzed a 1.3 million neural cell dataset within 30 min, obtaining 64 clusters which were mapped to 19 putative cell types. In particular, we further identified three different neural stem cell developmental trajectories in these clusters. We also classified two subpopulations of malignant cells in a small glioblastoma dataset using scAIDE. We anticipate that scAIDE would provide a more in-depth understanding of cell development and diseases.

## INTRODUCTION

The advancement in single-cell RNA-sequencing technology has grown exponentially in terms of sample sizes and accuracy (1–3). Identifying different cell types and sub-types remains one of the initial core analysis in single-cell data, prior to further downstream analysis. As the amount of data increases, we can gain a more holistic view of the identity and functionality of each cell. With recent large-scale pilot studies such as the Human Cell Atlas (4), unsupervised scalable and accurate computational approaches are essential for identifying different cell types.

Although many different computational methods have been developed to cluster and classify single-cell datasets, there is a trade-off between computational time and accuracy. Briefly, classification approaches are fast in analysis (provided that the model has been trained) with relatively reliable accuracy. However, such methods (5–7) require prior knowledge and labeled datasets for training (8). On the other hand, most clustering methods follow the pipeline of (i) clustering into groups, (ii) identify significantly expressed genes and (iii) manually validate marker genes with cell types. In general, there are three main categories of clustering methods. Traditional methods, including SC3, pcaReduce, Seurat, SIMLR and various others, rely on conventional dimensionality reduction methods such as PCA or t-SNE and then apply k-means or graph-based clustering to identify clusters (9–13). Secondly, iterative methods such as BackSPIN, SAIC and Panoview (14–16), attempt to provide a hierarchical structure over the identified cell clusters. More recent studies focus on developing deep learning methods to model the dropout events and provide nonlinear dimensionality reduction to represent single-cell datasets better (17–20). Despite the efforts in developing efficient and complex models, the clustering performance can be severely affected by hyper-parameters and varies accordingly in different datasets (21).

Identifying rare cell types is important in dissecting the cellular heterogeneity in global cell population. Recent methods, including RaceID, CellSIUS and GiniClust3, utilize clustering steps followed by an assignment step to identify rare cell types (22–25). In particular, CellSIUS first partitions the cells into coarse clusters and then identifies rare cell subpopulations based on correlated genes sets with respect to each subpopulation. The benefit of CellSIUS is that upregulated genes sets could be obtained for the identified cell types. Another recent method, FiRE (26), developed an algorithmic approach by directly assigning a rareness score to each cell without clustering.

**Figure 1.**
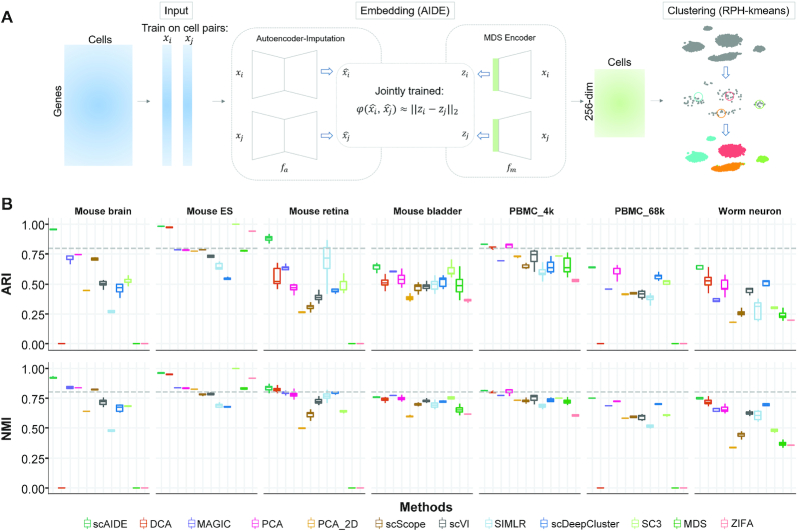
Overview and performance of scAIDE. (**A**) A schematic overview of the architecture of scAIDE. (**B**) The overall comparison of ARI and NMI performance on seven single-cell datasets. The dotted gray line represents a threshold of 0.8. For scDeepCluster and SC3, each algorithm was run five times to generate a distribution of results. For methods that involved a dimension reduction step, five embeddings were generated with the same parameters, and we obtained the boxplot by applying clustering to each embedding 10 times. We set k as the number of known cell type labels for comparison. Observations at 0 indicate that the experiment was not performed either because of insufficient memory or running time was >4 h.

In this manuscript, we propose a fully unsupervised deep learning clustering analysis framework, scAIDE (Figure 1A). First, we implemented an autoencoder-imputed distance-preserved embedding network (AIDE) to obtain a good representation of single-cell data which separates different cell types well. Subsequently, to identify small or rare cell types as well as common cell types, we developed a random projection hashing based *k*-means algorithm (RPH-kmeans). We can also automatically detect the number of clusters based on RPH-kmeans. Moreover, we provide a systematic biological analysis on the annotation of cell types. The performance and stability of scAIDE are extensively compared to existing state-of-the-art methods on seven single-cell datasets across different sequencing protocols. We further applied our clustering framework to analyze cell subpopulations in a small tumor dataset, a 68k peripheral blood mononuclear cells (PBMC), and finally, a 1.3 million neural dataset (27). We were able to identify small distinct cell populations, such as Cajal-Retzius cells (accounting for about 1.6% of the total population, expressing *Reln* and *Tbr1*), which are important to modulate early cortical patterning (28). In general, scAIDE is a scalable and efficient clustering framework which is consistent when applied to different single-cell datasets.

## MATERIALS AND METHODS

### Overview of scAIDE

There are two main components in scAIDE, namely AIDE for dimensionality reduction and RPH-kmeans for clustering, as shown in Figure 1A. Subsequently, we developed a general pipeline to provide biological analysis using marker genes and visualization of possible cell type development based on AIDE embedding.

Most clustering methods include a variable gene selection process to reduce the matrix to a reasonable size. However, to retain most information, we believe that the full gene expression should be used with minimal pre-processing (Supplementary Note VI and Table S25). We first filtered cells and genes with a minimum count of 1, followed by cell normalization and log transformation used in Scanpy and Seurat (11,29). As a result, most of the datasets contained about 10 000–20 000 genes after pre-processing (Table 1).

**Table 1.** Description of analyzed datasets

Datasets	No. of cells	No. of genes used	No. of cell types	Group size (min, max)	Technology (reads/cell)
Brain 1.3m	1 300 774	23 909	-	-	10× V2 (18 500)
Mouse brain	160 796	20 803	7	(1826, 74 539)	10× V1
PBMC 68k	68 579	20 387	10	(176, 21 429)	10× V1 (20 000)
PBMC 4k	4271	16 653	8	(135, 1292)	10× V2 (87 000)
Mouse bladder	2746	19 771	16	(13, 717)	Microwell-seq
Mouse retina	27 499	13 166	19	(48, 10 888)	Drop-seq (8200)
Mouse ES	2717	24 047	4	(303, 933)	Droplet-based
Worm neuron	4186	13 488	10	(70, 1015)	sci-RNA-seq
Jurkat 293T cells	1580	1000	2	(40, 1540)	10×
MGH107 (WHO II)	252	23 686	3	(4, 98)	Smart-seq2
Simulation datasets	5000	10 000	10	Approx. (68, 1099)	

### Autoencoder-imputed distance-preserved embedding (AIDE)

The architecture of AIDE consists of an imputation module and a dimension reduction module, as shown in Figure 1A. In the imputation module, the gene expression vector is fed to an autoencoder (AE) to correct biological noise such as dropout events. As AE captures the important latent structure of the data in the hidden layer and learns to regenerate the data, it is a natural extension that we can recover an imputed expression vector. We also added dropout layers in AE to avoid overfitting. Considering that the hidden vectors produced by simple AE may not be suitable for Euclidean-based clustering methods (e.g., *k*-means), we developed a fully connected network called multidimensional scaling (MDS) encoder in the dimension reduction module. The MDS encoder represents the dissimilarity/distance between the imputed expression vectors (produced by AE) as the Euclidean distance between projected points in a low-dimensional space. This matches with our latter developed random projection hashing based clustering algorithm which is also Euclidean-based.

Specifically, let $D\ = \ \{ {{\boldsymbol{x}}_i}\} _{i\ = \ 1}^N$ be the dataset with ${{\boldsymbol{x}}_i} \in {R^G}$ denoting the gene expression vector of cell *i*, *G* denotes the number of genes and ${D_p} = \ \{ ( {{{\boldsymbol{x}}_i},\ {{\boldsymbol{x}}_j}} )|{{\boldsymbol{x}}_i},\ {{\boldsymbol{x}}_j}\ \epsilon {\rm{\ }}D\}$ denotes all the cell pairs. The reconstruction loss of AE is defined as:

$$\begin{equation*}{L_{{\rm rec}}}\ = \frac{1}{{2\left| {{D_p}} \right|}}\mathop \sum \limits_{\left( {{{\rm{x}}_i},{{\rm{x}}_j}} \right) \in {D_p}} \left( {\left| {\left| {{{{\bf x}}_i} - {{{{\bf \hat{x}}}}_i}||_2^2 + } \right|} \right|{{{\bf x}}_j} - {{{{\bf \hat{x}}}}_j}||_2^2} \right),\end{equation*}$$

where ${\boldsymbol{\hat{x}}} = {f_a}\ ( {{\boldsymbol{x}};{{\boldsymbol{W}}_a}} )$ is also a *G*-dimensional vector with imputed gene expression, and ${{\boldsymbol{W}}_a}$ denotes the learnable weights of the AE, ${f_a}$. MDS (30) is a dimension reduction technique that preserves the dissimilarity/distance between pairs of objects. Here, we developed a neural network adaption of MDS to generate a low-dimensional representation of ${\boldsymbol{x}}$ by preserving the dissimilarity between ${\boldsymbol{\hat{x}}}$. The corresponding loss is defined as follows:

$$\begin{equation*}{L_{{\rm mds}}} = \frac{1}{{\left| {{D_p}} \right|}}\ \mathop \sum \limits_{\left( {{{\boldsymbol{x}}_i},{{\boldsymbol{x}}_j}} \right) \in {D_p}} \left| {||{{\boldsymbol{z}}_i} - {{\boldsymbol{z}}_j}||_2^2 - {\rm{\varphi }}{{\left( {{{{\boldsymbol{\hat{x}}}}_i},{{{\boldsymbol{\hat{x}}}}_j}} \right)}^2}} \right|\end{equation*}$$

where ${\boldsymbol{z\ }} = {f_m}\ ( {{\boldsymbol{x}};{{\boldsymbol{W}}_m}} )$ is a d-dimensional vector $(d \ll G)$, and ${{\boldsymbol{W}}_m}$ is the learnable weight of MDS encoder, ${f_m}.\ \,{\rm{\varphi }}$ denotes a specific dissimilarity/distance metric in the space of the imputed gene expression, and we used the Euclidean distance in this paper:${\rm{\varphi \ }}( {{{{\boldsymbol{\hat{x}}}}_i},{{{\boldsymbol{\hat{x}}}}_j}} ) = \ ||{{\boldsymbol{\hat{x}}}_i} - {{\boldsymbol{\hat{x}}}_j}|{|_2}$.

The training of AIDE can be divided into two stages: pre-training and joint tuning. In the pre-training stage, parameters of AE are optimized by minimizing ${L_{{\rm rec}}}( {{D_p};{{\boldsymbol{W}}_a}} )$. In the joint tuning phase, both the AE and MDS encoder are trained by minimizing

$$\begin{equation*}L\ \left( {{D_p};{{\boldsymbol{W}}_a},{{\boldsymbol{W}}_m}} \right) = {L_{{\rm rec}}}\ \left( {{D_p};{{\boldsymbol{W}}_a}} \right) + {\rm{\alpha }}{L_{{\rm mds}}}\left( {{D_p};{{\boldsymbol{W}}_a},{{\boldsymbol{W}}_m}} \right),\end{equation*}$$

where ${\rm{\alpha }} >\ 0$ is the coefficient that controls the relative weights of ${L_{{\rm rec}}}$ and ${L_{{\rm mds}}},$ which also affects the degree of fitness of AE. Note that there is no need to generate all possible combinations of cell pairs $({D_p})$ for AIDE training. In practice, we feed the model with a mini-batch of cell pairs randomly selected from dataset *D* iteratively until the training converges. After training, we use the MDS encoder to generate the embeddings $\{ {{\boldsymbol{z}}_i}\} _{i\ = \ 1}^N,$ taking the gene expression vectors $\{ {{\boldsymbol{x}}_i}\} _{i\ = \ 1}^N$ as input.

We further compared the performance of AIDE against single components of AE and MDS encoder to show the outperformance of the novel architecture (Supplementary Figure S1, Note II and Tables S12–13).

### Random projection hashing-based *k*-means clustering (RPH-kmeans)

As k-means is simple with low time complexity, many studies adapt it to cluster single-cell RNA-seq data (9,10,22). However, one major downside is that k-means is highly sensitive to initial cluster centers. Thus, when the size of the underlying cluster groups is highly imbalanced, which is often the case with single-cell data (Table 1), the resulting clusters become biased toward larger cell populations. The standard random initialization and the most popular *k*-means++ (31) initialization strategy both choose initial centers located in large-size groups with extremely high probability. As a result, the cluster centers will be stuck in large groups since small groups have little impact on calculating new centers during each iteration. The larger groups may eventually be partitioned into several parts, leading to poor clustering performance. (Supplementary Figure S2).

In order to solve the data imbalance problem, we proposed a random projection hashing based k-means termed RPH-kmeans, which initializes the cluster centers using one of the locality sensitive hashing (LSH) (32) techniques. The key principle of LSH is to project close data points to the same bucket with high probability. D. Datar *et al.* (33) proposed an LSH family for ${l_p}$ distance metric. When *p* is 2 (the distance between two data points is evaluated by the Eulidean metric), the random projection-based hashing (RPH) function that maps a data point ${\boldsymbol{v}} \in {R^d}$ to an integer is defined as:

$$\begin{equation*}{h_{{\boldsymbol{a}},b}}\ \left( {\boldsymbol{v}} \right) = \left\lfloor {\frac{{{\boldsymbol{a}} \cdot {\boldsymbol{v}} + b}}{w}} \right\rfloor ,\end{equation*}$$

where ${\boldsymbol{v}} \in {R^d}$ denotes a data point, ${\boldsymbol{a}} \in {R^d}$ is a random vector with ${a_i}$ drawn i.i.d. from the standard Gaussian distribution $N( {0,1} )$, *b* is a random variable drawn from the uniform distribution $U( {0,w} )$, and *w* denotes the quantization step. Next, a composite hash function $g( {\boldsymbol{v}} )$ is constructed by combining *l* hash functions:

$$\begin{equation*}g\ \left( {\boldsymbol{v}} \right) = \left( {{h_1}\left( {\boldsymbol{v}} \right), \cdots ,{h_l}\left( {\boldsymbol{v}} \right)} \right)\ \end{equation*}$$

Thus, given a data point **ν**, the LSH function *g* will project **ν** to an integer hash code vector. Data points are considered to be hashed into the same bucket if their hashed code vectors are exactly the same. In general, the closer (evaluated by the Euclidean distance) two data points are, the more likely they will be hashed into the same bucket.

The pipeline of cluster center initialization of RPH-kmeans can be summarized in two phases. In the first phase, the number of data points is reduced iteratively using LSH. In each iteration, the data points hashed to the same bucket will be merged to a weighted point. Finally, a data skeleton with much fewer points is generated. In the second phrase, weighted *k*-means (Algorithm S2) with *k*-means++ initialization will be applied to the skeleton to produce initial centers for RPH-kmeans. Since the number of points in large groups is significantly reduced, potential bias caused by data imbalance can be alleviated (as shown in Supplementary Figure S3). The pseudocode for RPH-kmeans initialization is described in Algorithm 2, and the full RPH-kmeans algorithm is described in Algorithm 1.

Due to the random property/character of LSH, error may be induced when generating the skeleton. For example, data points belonging to two large close groups are likely to be hashed into the same bucket, resulting in a poorly represented skeleton. Here, we provide two optional bucket correction strategy to solve the problem. Inspired by DACE (34), we first developed a radius-based strategy (Algorithm S3). Each bucket will be divided into sub-buckets by successively assigning every point to its closest center. If the distance to the center is greater than a given radius r, a new center will be created. The other one is called a size-based strategy (Algorithm S4). It retains only partial buckets with a small size because large buckets usually lead to the potential merging of two different groups.

Based on the weighted skeleton points generated by random projection, we further developed a weighted Bayesian Information Criterion (BIC) approach to estimate the number of clusters in the dataset (Supplementary Note I, Table S1, and Figure S4).

We noticed that the framework proposed by Li *et al.* (35) is similar to RPH-kmeans. However, they focused on using LSH to speed up k-means. To the best of our knowledge, we are the first to use LSH to approach the data imbalance problem in clustering.


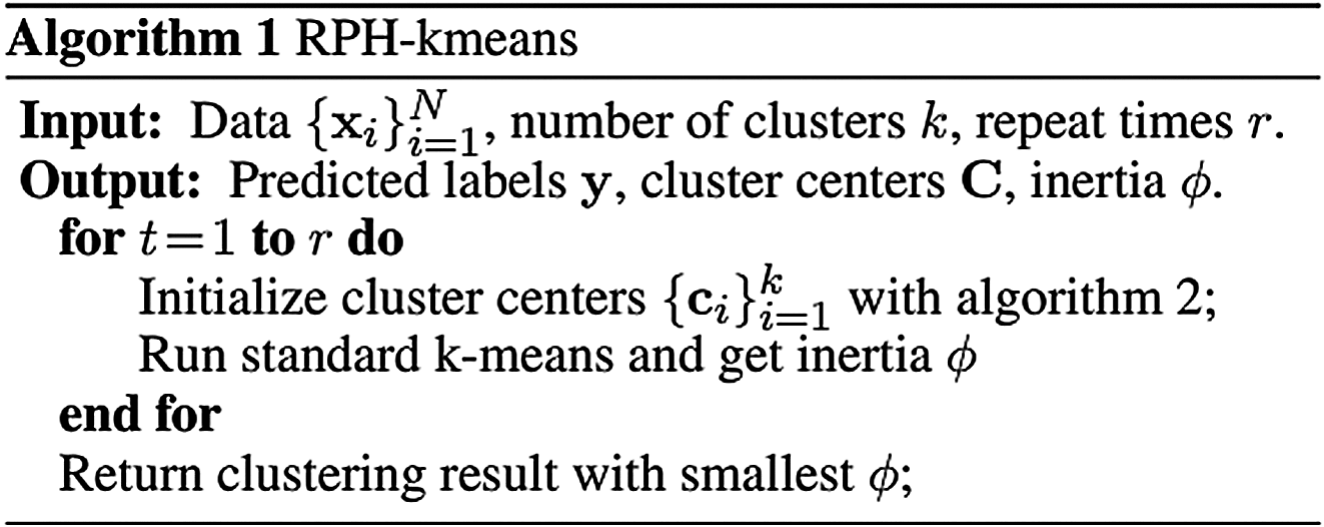



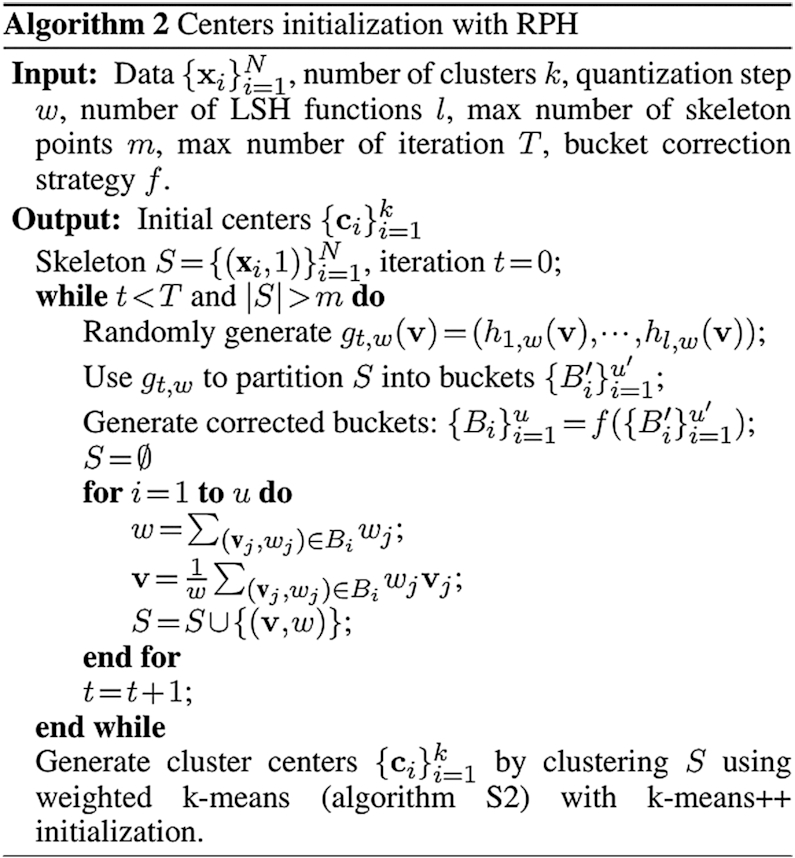


### Evaluation metrics

All clustering results are measured by the adjusted rand index (ARI) (36) and normalized mutual information (NMI) (37). Given two partitions *U* and *V*, let ${n_{ij}}$ be the number of common data points of groups ${u_i} \in U$, and ${v_j} \in V$. ARI is defined as:

$$\begin{equation*}{\rm ARI}\ = \frac{{\mathop \sum \nolimits_{i,j} \left( {\begin{array}{@{}*{1}{c}@{}} {{n_{ij}}}\\ 2 \end{array}} \right) - \mathop \sum \nolimits_i \left( {\begin{array}{@{}*{1}{c}@{}} {{n_{i \cdot }}}\\ 2 \end{array}} \right)\mathop \sum \nolimits_j \left( {\begin{array}{@{}*{1}{c}@{}} {{n_{ \cdot j}}}\\ 2 \end{array}} \right)/\left( {\begin{array}{@{}*{1}{c}@{}} n\\ 2 \end{array}} \right)}}{{\frac{1}{2}\left[ {\mathop \sum \nolimits_i \left( {\begin{array}{@{}*{1}{c}@{}} {{n_{i \cdot }}}\\ 2 \end{array}} \right) + \mathop \sum \nolimits_j \left( {\begin{array}{@{}*{1}{c}@{}} {{n_{ \cdot j}}}\\ 2 \end{array}} \right)} \right] - \mathop \sum \nolimits_i \left( {\begin{array}{@{}*{1}{c}@{}} {{n_{i \cdot }}}\\ 2 \end{array}} \right)\mathop \sum \nolimits_j \left( {\begin{array}{@{}*{1}{c}@{}} {{n_{ \cdot j}}}\\ 2 \end{array}} \right)/\left( {\begin{array}{@{}*{1}{c}@{}} n\\ 2 \end{array}} \right)}}\ \end{equation*}$$

where ${n_{i \cdot }} = \mathop \sum \limits_j {n_{ij}}\ ,\ {n_{ \cdot j}} = \mathop \sum \limits_i {n_{ij}}$.

NMI is defined as:

$$\begin{equation*}{\rm NMI}\ = \frac{{\mathop \sum \nolimits_{i,j} \frac{{{u_i} \cap {v_j}}}{n}\log \frac{{n\left| {{u_i} \cap {v_j}} \right|}}{{\left| {{u_i}} \right|\left| {{v_j}} \right|}}}}{{\frac{1}{2}\left( { - \mathop \sum \nolimits_i \frac{{\left| {{u_i}} \right|}}{n}\log \frac{{\left| {{u_i}} \right|}}{n} - \mathop \sum \nolimits_j \frac{{\left| {{v_j}} \right|}}{n}\log \frac{{\left| {{v_j}} \right|}}{n}} \right)}}\ \end{equation*}$$

where *n* is the number of data points.

### Data visualizations and biological analysis

In order to visualize the distribution of cluster groups and the embedding of scAIDE, we used t-stochastic neighboring embedding (t-SNE) for all our visualizations. The default parameters are applied without tuning using the R package, Rtsne.

For the discovery of marker genes, we first calculated the Wilcoxon's rank-sum test for each gene in the cluster. Then the log fold change values were measured to ensure that the identified marker gene is supported by sufficient samples. The threshold cut-off for the rank-sum test is set to a small value near 0 (for a strict detection of a small number of marker genes) and 1.5 for fold-change. Fold-change values were calculated as the ratio between group average gene expressions. We are only interested in the up-regulation of markers within a specific cluster, compared to the remaining cells.

In some current studies, cell types are assigned according to a few top marker genes. We believe that developing a systematic approach to assign cell types would be more reliable. To classify the cell types in the clustering analysis, we use gene markers from previous studies (38) and a single-cell gene marker database (39). We used a simple matching rate and the Jaccard index to quantify the number of overlapping marker genes. To test the significance of the assigned cell type, we implemented an enrichment *P*-value based on a hypergeometric distribution (40). After filtering, we define the total number of genes, *G* as the number of background genes. Suppose α denotes the number of identified markers from a particular cluster, and *b* the number of markers for a specific cell type, the number of overlapping genes is regarded as *k*. The enrichment *P*-value was calculated as follows:

$$\begin{equation*}p\ = \ \mathop \sum \limits_{i\ = \ k}^{{\rm{min}}\left( {a,\ b} \right)} \frac{{C_i^a.C_{b - i}^{N - a}}}{{C_b^N}}\end{equation*}$$

Additionally, manual validations were also made by comparing specific top markers with existing studies.

Identifying possible trajectory development is one of the important downstream analysis in single-cell data. We utilize the AIDE embedding vectors to reflect the development of cell clusters. The intuition behind this is that cells from a similar lineage would be closer. First, we calculate the average AIDE vector for each respective cluster, and then we apply the Euclidean distance to clusters to obtain a *k* by *k* matrix, where *k* is the number of clusters. Then we perform a simple hierarchical clustering (with complete linkage) to reflect the relationship between cell clusters. Finally, we visualize the cell clusters using heatmap and dendrogram to depict the groupings of possible trajectory development.

### Datasets

#### Real datasets

We used a total of 9 real single-cell datasets to quantify the performance of scAIDE in clustering analysis. The summary of each dataset is listed in Table 1, spanning across different sequencing technologies and varying dropout rates.

#### Simulation datasets

Concerning the rate of dropout events in more efficient sequencing technologies, it is necessary to develop robust clustering methods that can be generalized. We compared the current methods on multiple simulated datasets using splatter (41), ranging from 60% of dropouts to 93% dropouts (hereby referred to as sparsity). We simulated single-cell datasets with 5000 cells and 10 000 genes, with a highly imbalanced cell group assignment. The smallest cluster contained about 1.5%, while the largest contained roughly 23.2% of cells. Simulation parameters were obtained from a pre-processed smart-seq2 single-cell dataset of the development in mouse embryonic cells (42). The sparsity was tuned by altering the dropout parameters in splatter. The performance of the algorithms is evaluated using pre-determined group labels.

Additionally, we simulated another mouse embryonic dataset (43) by increasing the dropout sparsity of the dataset. We followed the pipeline of simulating dropout as used in splatter. The dataset had an original sparsity of about 70%; then, we simulated the data for 85, 90 and 96%.

## RESULTS

### Quantitative evaluation of scAIDE

To benchmark the general performance of scAIDE, we compared it to multiple state-of-the-art methods. This includes simple baselines such as MDS, PCA and PCA_2D followed by *k*-means, complex approaches like SC3 (9), SIMLR (12), MAGIC (44) and numerous deep learning methods: DCA (17), scDeepCluster (19), ZIFA (20), scVI (45) and scScope (46) (Figure 1B). One interesting observation was that PCA performed well when the gene expression was reduced to 256 components (PCA) instead of two components (PCA_2D) before applying the k-means clustering. Although PCA_2D (with two components) is commonly used as the baseline in many studies, we argue that significantly better results could be achieved by simply increasing the number of components (which better captures the information of the data). Thus, we hypothesized that a good representation of the gene expression data might lead to profound biological insights, even with simple clustering methods.

We used ARI and NMI to quantify how similar the clustering results are to the cell labels given in their respective original studies. A total of seven single-cell datasets were used to evaluate the performance, ranging from very distinct cell populations (for example, mouse embryonic stem cells) to very diverse populations (such as neural cells and PBMCs). Although some labels were not of the gold standard, these datasets provide a general baseline to compare the performance of current state-of-the-art clustering methods. scAIDE first reduces the gene expression input to a reduced AIDE embedding of 256-dimensions, followed by our developed RPH-kmeans clustering approach. In order to maintain a fair comparison for deep learning methods, we followed their pre-processing steps for each respective method. Also, we tested both their default embedding dimensions, and parameters which had similar model complexity to AIDE (Supplementary Note III and Tables S14–21). We plotted the best results and compared their performance in Figure 1B and Supplementary Note III. Overall, scAIDE demonstrates high stability and overall performance over current methods. In particular, our method outperformed other methods significantly in the mouse retina dataset (47), which contained 19 cell types with a total of 27 499 cells. Although it is highly imbalanced (the smallest group only contained 48 cells), our approach achieved a high average of 0.875 ARI and 0.825 NMI. Additionally, we also show that RPH-kmeans improves the clustering performance by benchmarking against *k*-means++ and *k*-means (with random initialization) on these seven datasets (Supplementary Note IV and Table S22).

We kept a small number of parameters for convenience and provided default parameters which demonstrate consistent performance across datasets (Supplementary Figure S5 and Table S2). We demonstrate that scAIDE is relatively stable using default parameters with little tuning required; the details for the parameters used in the experiments are included in the supplementary materials (Supplementary Tables S2-S6 and Note III).

### Robustness of scAIDE under different dropout simulations

Although more accurate sequencing technology is emerging, cost and efficiency still constrain deep sequencing. Scalable technology, such as 10×, sequences at an average of 10–20k reads per cell, while smart-seq2 can sequence up to millions of reads per cell but at a higher cost. Thus, it is vital that computational methods can accurately recover the cell-type populations at various dropout rates.

**Figure 2.**
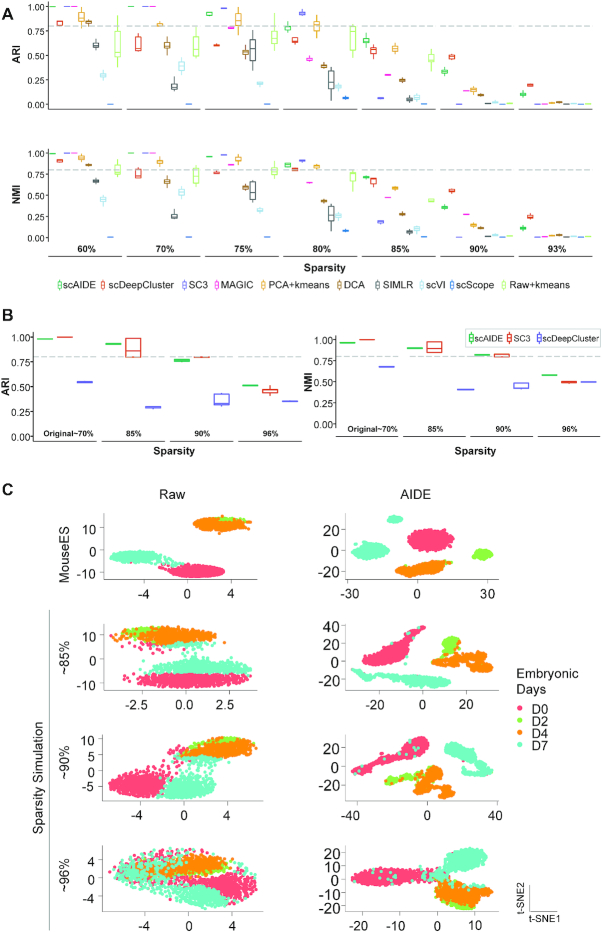
Dropout simulation and analysis. (**A**) Fully simulated single-cell datasets were generated using *splatter*, ranging from 60% sparsity to 93%. Boxplots follow similar settings to Figure 1B. We used the default parameters for scAIDE and set the maximum training steps to 40 000 without early stop. (**B**) Simulations were obtained by adding dropout events to the mouse ES dataset, increasing from 70% to about 96%. We used the default parameters for scAIDE. The left panel shows the ARI performance of scAIDE, SC3 and scDeepCluster, similarly for NMI on the right. The boxplots were drawn by running each algorithm five times and obtaining a distribution. (**C**) t-SNE visualizations of the raw gene expression matrix and the AIDE representation of the mouse ES dataset. Colors represent the true cell labels.

We first evaluated the performance on fully simulated datasets using splatter (41), ranging from roughly 60% of zero expressions (similar proportion as seen in smart-seq2 datasets) to 93% (similar to 10× datasets). We simulated 5000 single-cell profiles of 10 000 genes, with 10 imbalanced cell groups (Figure 2A). In cases of lower sparsity levels (60–85%), the performance of scAIDE was on par with the consensus and imputation methods, SC3 and MAGIC; while outperforming other deep learning methods. scDeepCluster performed well in the two simulation cases with higher dropout rates (90 and 93%), despite its moderate performance on real datasets. This could be due to the use of a zero-inflated negative binomial (ZINB) layer in its model.

In order to further validate this result, we generated more realistic simulations by adding dropout events based on a logistic regression model (41). We used a well-defined reference dataset that consists of mouse embryonic stem cells (Mouse ES (43)) with four leukemia inhibitory factor (LIF) withdrawal time interval labels (day 0, 2, 4 and 7). SC3 was used as a baseline for comparison due to its superior performance on this dataset. We also included scDeepCluster as it performed relatively well in the previous simulation experiments. In Figure 2B, each algorithm was run five times, and their respective performance indexes were plotted. Despite the good performance of scDeepCluster in the previous simulated datasets, it fails to separate the cells here. scAIDE is highly consistent in all scenarios and outperforms SC3 when sparsity level exceeds 90%. Furthermore, the AIDE embedding clearly separates cells from the four different intervals, even in cases of high dropout events (Figure 2C). We show that scAIDE has a high potential to separate and delineate cell groups regardless of dropout situations.

### Identification of putative and rare subpopulations in single-cell datasets

*De novo* clustering analysis has the potential to provide biological insight into the identification of rare cell types. In general, there are two important aspects in accurately separating different cell types and identifying rare subpopulations. First, cells should be well-represented in low dimensions; subsequently, clustering algorithms should accommodate the identification of small groups of cells. In simulation experiments, we first depict that the AIDE embedding is capable of separating different cell types, and that RPH-kmeans is tailored for the detection of rare cell types. Then, we applied scAIDE to three different datasets (case studies) to reveal novel biological findings. Particularly, not only did we identify different subpopulations within each dataset, but we also identified primed differentiation development of cell types.

#### Rare cell type detection in simulated datasets

**Figure 3.**
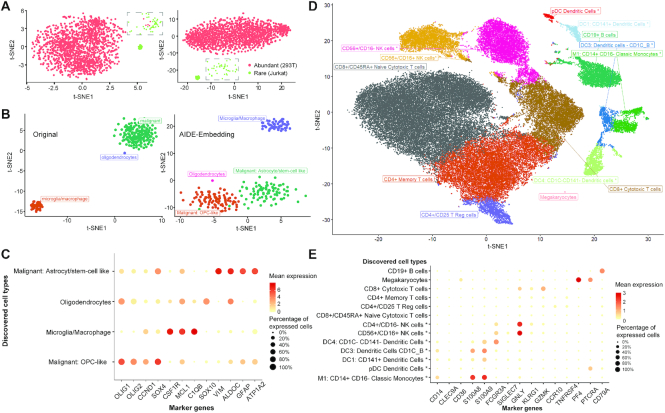
Deciphering cell subpopulations from global clustering results. (**A** and **B**) t-SNE plots showing the normalized gene expression profile with cell labels on the left, and t-SNE after AIDE embedding on the right with annotated labels determined from clustering results. (A) Simulated experiment on mixing 2.5% of the rare population (Jurkat cells in green) with an abundant population (293T cells in red). (B) A labeled glioblastoma dataset with 252 cells. OPC: oligodendrocyte progenitor cells. (**C**) Significantly expressed marker genes in the tumor dataset. The size of the points represents the percentage of cells that expressed the particular marker within the specific cell type. Color depicts the average log normalized expression. (**D** and **E**) Analysis of PBMC 68k dataset. Annotations with an asterisk (*) imply that they are new findings of cell subtypes. (D) t-SNE visualization of AIDE embedding with annotated labels. (E) Significantly expressed markers genes according to respective cell types within PBMC dataset.

Following Zheng's study (3) and a rare cell type detection method named FiRE (26), we mixed 2.5% (40 cells) of Jurkat cells into an abundant population of 293T cells, totaling to 1580 cells. Using a pre-processed and normalized expression containing 1000 filtered genes, we evaluated the performance of scAIDE (Figure 3A). According to the original publication and reproduced results, FiRE achieved an F1-score of 0.71 with 32 false positives. Using the clustering setting, we set *k* = 2 and obtained an F1-score of 1.0 for scAIDE (with default parameters), while SC3 achieved 0.94 with five false positives. CellSIUS also achieved an F1-score of 1.0, and GiniClust3 (parameter neighbors set to 10) achived a high score of 0.97 with two false positives. As depicted on the left panel of Figure 3A, five 293T cells (red) were well-mixed into the rare subpopulation of Jurkat cells (green). The AIDE embedding precisely delineates the subpopulation from the abundant group (right panel of Figure 3A). As mentioned in a previous study (24), clustering methods may perform poorly when the rare subpopulation percentage drops below 2%. We compared scAIDE with CellSIUS, GiniClust3 and FiRE for 0.5, 1, 1.5 and 2% mixtures of Jurkat cells (Supplementary Figure S10). scAIDE was able to separate the rare cell type (Jurkat cells) exactly in all four cases (Supplementary Figure S11).

To address the imbalanced composition of cell types, we further simulated rare cell type composition in datasets. We retained the largest two cell types and then sampled 50 or 500 (depending on the size of the dataset) cells for each of the remaining cell types. If the number of cells was less than the sampling number, we retained all cells of that particular cell type. RPH-kmeans is extensively compared with *k*-means++ and *k*-means (random initialization) to show its outstanding capability in detecting rare cell type subpopulations (Supplementary Figure S6, Note V and Tables S23–24). For both PCA and AIDE dimensional reduction techniques, RPH-kmeans (default parameters) outperformed the others in detecting small clusters (evaluated by ARI and NMI). Specifically, on PCA embeddings of the PBMC 68k dataset (Supplementary Figure S6a), RPH-kmeans achieved an ARI of 0.544 with only one initialization while traditional *k*-means++ algorithm only achieved 0.343 (with 100 initializations). On the mouse retina dataset (Supplementary Figure S6b), where the composition of cell types is highly imbalanced, RPH-kmeans also achieved significantly better clustering results (ARI = 0.88 with 1 initialization) than k-means++ (ARI = 0.437 with 100 initializations). Thus, these results reflect that RPH-kmeans can identify rare subpopulations more accurately.

#### Case study I: Subpopulations of malignant cells in glioblastoma

Next, we applied scAIDE to a glioblastoma dataset (MGH107) (48). It is important to understand and dissect the underlying cell types in tumor micro-environments (TME). This dataset contains only 252 cells, and we were able to identify subpopulations in malignant cells (Figure 3B) using AIDE. As analyzed in one of our previous work (49), a subset of malignant cells express astrocytic and stem-cell-like genes (*GFAP*, *ALDOC*, *ATP1A2*, *VIM*), resembling the results shown in Figure 3B and C. Furthermore, we classified the second subset of malignant cells to be oligodendrocyte progenitor cell-like (OPC-like), expressing *OLIG1*, *OLIG2*, as well as proliferative markers such as *SOX4* and *CCND2*. Additionally, discovered markers can be found in Supplementary Table S7 (*P*-value < $1\ \times {10^{ - 10}}$). The results demonstrate that malignant cells within TME have the potential to proliferate. By global unsupervised clustering analysis, we were able to identify a subgroup of malignant cells, adding to the understanding of development in tumors.

#### Case study II: Identification of common and rare subtypes in PBMCs

Subsequently, we applied our method to a larger dataset, containing about 68 000 PBMC cells (3). The original study reveals 11 cell types by calculating the correlation between reference transcriptomes to single-cell expressions. We first applied AIDE to generate a reduced embedding of the PBMC data (Figure 3D). We used weighted BIC (Methods) to automatically determine the number of clusters from the embedding (*k* = 13, Supplementary Figure S4a). Cluster-specific marker genes were cross-referenced with known markers (38) to annotate each cell type (‘Materials and Methods’ section). As a result, we identified subpopulations of natural killer (NK) cells, dendritic cells (DC), and monocytes. In particular, CD56+ CD16+ NK cell expresses *SIGLEC7* and *GNLY*, while CD56+ CD16- NK cell expresses *KLRG1* (50). Furthermore, four different subpopulations of DCs were identified as plasmacytoid DC (pDC), inflammatory DC, CD141+ DC and CD1C- CD141- DC. Some of their respective top markers are shown in Figure 3E. As t-SNE components may not fully capture the global structure of data (51), we developed a simple approach (Methods) to visualize and identify possible development trajectory of cell clusters (Supplementary Figure S8). Interestingly, cell clusters of the lymphoid progenitor lineage (T cells, B cells and NK cells) are clearly separated from those of the myeloid progenitor lineage (dendritic cells, monocytes and megakaryocytes). Notably, we identified the rare cluster of megakaryocytes (about 0.24% of profiled cells) and the subpopulations of dendritic cells (ranged from 0.5 to 2.3% of the profiled population), similar to the results reported by the authors of FiRE (26).

#### Case study III: Cell type decomposition and primed differentiation process in a 1.3m neural dataset

**Figure 4.**
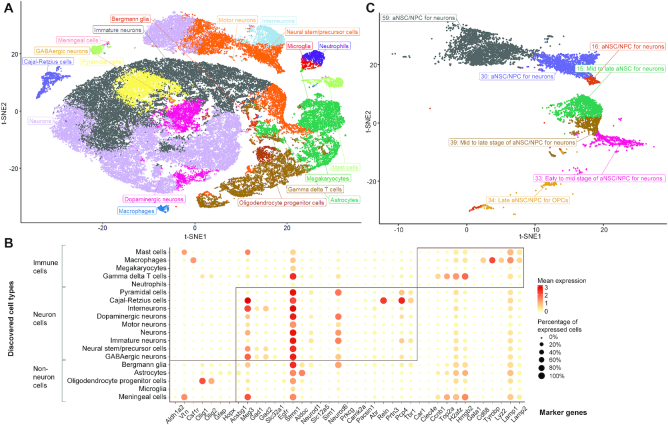
Putative and rare cell type discovery in the 1.3 million neural cell dataset. (**A**) t-SNE visualization of the AIDE embedding on 5% sampled data from the full dataset. Data are sampled based on the 64 clusters obtained from scAIDE. Colors represent the 19 cell type annotations from our biological analysis. (**B**) t-SNE visualization on cells annotated with the label: neural stem/precursor cells. These correspond to the orange cells in the same position as in A. Annotations were validated by different markers and their respective positioning. NPC: neural progenitor cells; aNSC: activated neural stem cells; OPC: oligodendrocyte progenitor cells. (**C**) Top significantly expressed marker genes in each respective cell types.

Finally, to investigate the underlying biological insights in neural brain development, we applied scAIDE to provide a global clustering analysis on a 1.3 million neural dataset (27). Within 30 min, we obtained our embedding and 64 cluster assignments (Figure 4A and Supplementary Figure S4b). Using a curated list of markers from PanglaoDB (39), including both markers of neural and the immune system, we eventually mapped the 64 clusters to 19 putative cell types by their respective marker genes (Figure 4B). We further validated their cell types with an enrichment *P*-value (*P*-value < 0.05), shown in Supplementary Tables S8 and 9, except for the cluster of megakaryocytes which significantly expressed *Gata1*. Neuronal markers such as *Meg3* separates neuron cells from the rest of the cells. *Stmn1*, which had been reported to be highly expressed in later stages of neurogenesis (52), is consistently expressed throughout most neural cells (Figure 4B). Together, these results show that our clustering approach is capable of identifying putative cell types.

To further define cell subpopulations, we focus on the population of neural stem/precursor cells (orange cells shown in Figure 4A). A total of seven clusters were mapped to this cell type from global clustering (ranging from ∼0.28 to 2.4% of the total population). Hence, we attempted to delineate the different sub-types of neural stem cells or neural precursor cells. First, we visualized the separation of different cell clusters in a heatmap (Supplementary Figure S9) and identified three possible neural stem cell development based on AIDE embedding. Then, we utilized quiescent markers (*Clu*, *Id3*), activation markers (*Egfr*, *Atp1a2*, *Gfap*, *Prom1*), neurogenesis markers (*Dlx1*, *Dlx2, Dcx*, *Dlx6as1*) and astrocytic markers (*Fgfr3*, *Gja1*, *Jag1*) (53), to assign possible stages of cell types to each cluster (Figure 4C and Supplementary Tables S10–11). Interestingly, cluster 33 significantly expressed *Clu*, suggesting that a small portion of quiescent cells may be present. Since cluster 33 is clustered with OPCs and astrocytes (Supplementary Figure S9), we believe that these are early- to mid-aNSCs primed for OPCs (significantly expressed in astrocytic markers with *P*-value < 0.05). Clusters 16, 30 and 59 distinctively express neurogenesis markers, suggesting a cell fate toward interneurons and neurons. For cluster 34, although it groups with gamma delta T cells, oligodendrocyte markers of *Pdgfra* and *Olig2* are significantly expressed, suggesting an OPC lineage. The last two groups of mid- to late-neural stem/progenitor cells (clusters 15 and 39) are grouped between gamma delta T cells and neuronal cells (Figure S9). Although gamma delta T cells respond to neuroinflammation, a recent study shows that they promote short-term memory by controlling synaptic plasticity in hippocampus neurons at steady-state (54). The organization of our identified cell clusters also suggests a supportive role of gamma delta T cells within neuronal cell types. In conclusion, we were able to define detailed cell subpopulations in single-cell datasets using the scAIDE clustering analysis framework.

### Scalability

**Figure 5.**
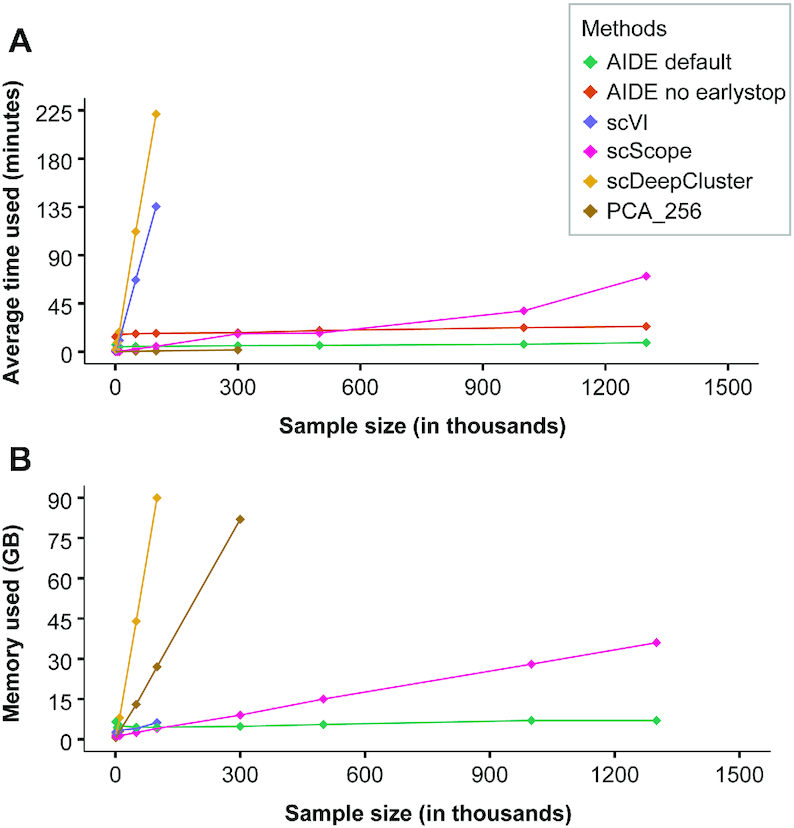
Comparison of computational efficiency. (**A** and **B**) Both graphs share the same legend, with (A) showing the computational time used in minutes and (B) depicting the amount of memory usage (in gigabytes, GB) for each method. As AIDE default and AIDE (no early stop) run with the same memory usage, we only plotted AIDE default in B.

In Figure 5, we compared the scalability and efficiency of AIDE against the embedding step in current deep learning methods (except for scDeepCluster which directly generates the clustering result). Different sample sizes were sampled from the 1.3 million single-cell dataset to assess their performance, where the number of genes remains consistent. All experiments were performed on our CentOS system with 24 CPU cores at 2.5GHz, 125GB of memory and one 1080Ti graphics card. By default, AIDE reduces the data to 256 dimensions. The number of reduced dimensions were shown on the plot for all other methods. It should be noted that scScope pre-processed the data by selecting only the top 1000 variable genes as input to their algorithm. All other methods had the input of the full gene expression profile. Even with early stop disabled, we were able to obtain an AIDE embedding within 24 min using only 7 GB of memory on the full 1.3 million single-cell dataset.

Additionally, our clustering algorithm (RPH-kmeans) not only improves the detection of rare populations but is also more efficient than *k*-means++ or *k*-means (with random initialization) in some cases; where better-initialized centers lead to faster convergence and better result (Supplementary Figure S7). Thus, scAIDE is a highly efficient approach for analyzing huge single-cell datasets.

## DISCUSSION

To date, it has been of great interest to use single-cell sequencing technology to identify both common and rare cell types in complex tissues. Most common cell types have been discovered long and are well-studied; however, rare sub-populations often remain obscure, particularly in diseases. Typical clustering approaches may be limited in both their ability to identify minor populations and computational time (55). We developed scAIDE to provide accurate and efficient clustering analysis, delineating both putative and rare cell types in single-cell datasets.

While many deep learning-based methods have been developed (17,19), including scalable methods such as scScope (46), we show that their performance is inconsistent between simulations and real datasets. Our analysis demonstrated the robustness of scAIDE in cases of high dropout rates and its ability to delineate rare cell types. In most cases, default parameters or small tuning would be sufficient.

In particular, we analyzed a small tumor dataset with scAIDE, identifying important sub-populations of malignant cells that express different lineage markers. Additionally, scAIDE was able to cluster rare cell types of megakaryocytes and dendritic subpopulations (ranging from 0.24 to 2.3% of profiled cells) in the PBMC 68k dataset. We also showed its ability to determine clusters which were correctly assigned to putative cell types (with significant enrichment p-values) in a 1.3 million neural cell dataset. Three different lineage development branches were identified by further investigation of seven clusters assigned to the neural stem/progenitor cells. Together, we demonstrate the capability of scAIDE to reveal putative and rare cell types in single-cell datasets. We believe that there is excellent potential for scAIDE to be further incorporated into trajectory development analysis in the future.

Within only 30 min, scAIDE could cluster the 1.3 million single-cell dataset using only 7 GB of memory. Together with its consistent performance and downstream biological analysis, we believe that our clustering analysis framework would provide a deepened understanding of cell types and developments within complex tissues and diseases.

## DATA AVAILABILITY

scAIDE is publicly available via https://github.com/tinglabs/scAIDE.

## Supplementary Material

lqaa082_Supplemental_Files

## DECLARATION

The funders had no roles in study design, data collection and analysis, the decision to publish and preparation of the manuscript.

## Notes

Present address: Department of Computer Science and Technology, Tsinghua University, Beijing, China.
