# Supplementary material for: scAIDE: clustering of large-scale single-cell RNA-seq data reveals putative and rare cell types
Source: NAR Genom Bioinform. 2020 Oct 9;2(4):lqaa082. doi: 10.1093/nargab/lqaa082 (PMC7671411; doi:10.1093/nargab/lqaa082)
Supplement: lqaa082_Supplemental_Files [file lqaa082_supplemental_files.zip › Supplementary Materials.docx]

**Supplementary Materials**

**Manuscript title:**

**scAIDE: Clustering of large-scale single-cell RNA-seq data reveals putative and rare cell types**

**Benchmarks on different components of AIDE architecture:**


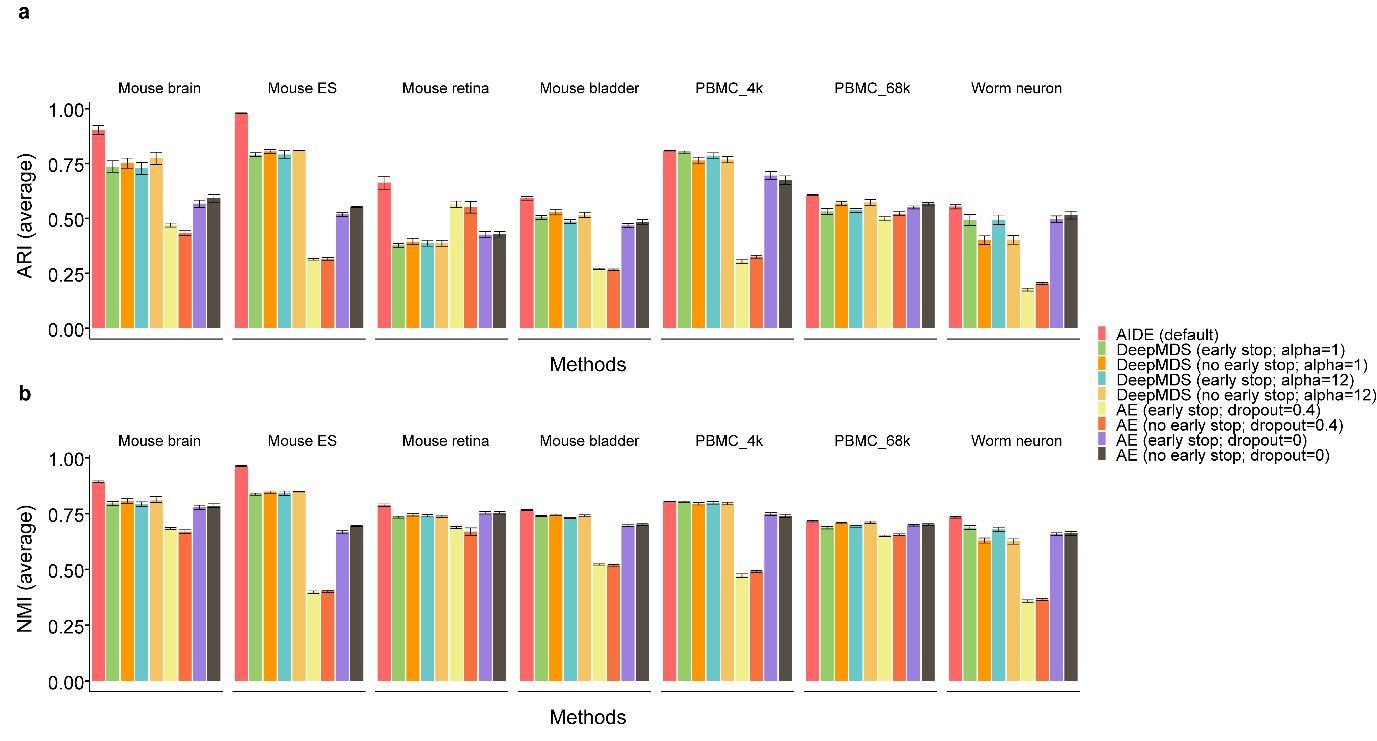


**Figure S1.** Benchmark comparisons of different parts of AIDE. AIDE is jointly trained on an autoencoder-imputation model (AE) and MDS encoder. Here we show the results for each separate part, compared against AIDE (default parameters) in 7 benchmarking datasets. **a)** Results shown for ARI. **b)** Results shown for NMI. Error bar represents the 95% confidence interval based on 50 clustering results (10 k-means++ clustering results were recorded for each of 5 generated embeddings, using respective parameters).

**Simulation experiments on RPH-kmeans:**

Using 5 different 2-D Gaussian distributions, we generated a simulation dataset with 5,000, 100, 100, 100, and 100 data points for each respective parameter. The goal of this experiment is to show the deficiency in current k-means initialization methods when the data labels are highly imbalanced. Results for k-means++ and random initializations are shown in Figure S2, and RPH-kmeans shown in Figure S3.


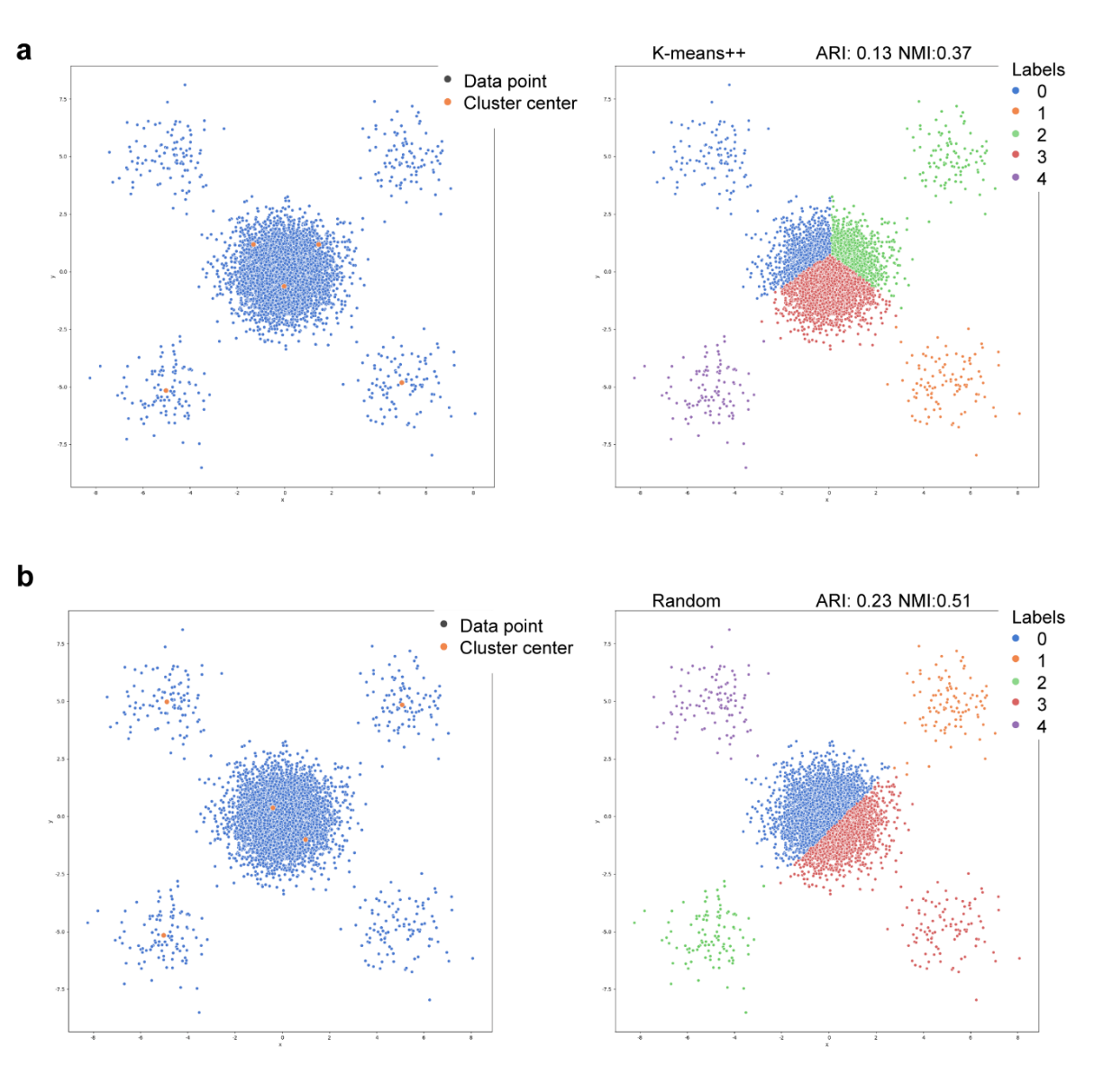


**Figure S2.** Results for simulation of 2-D data using k-means++ and k-means with random initialization. **a)** The final cluster centers identified by k-means++ is shown on the left, and clustering results on the right. **b)** The final cluster centers identified by k-means with random initialization is shown on the left, and clustering results on the right.


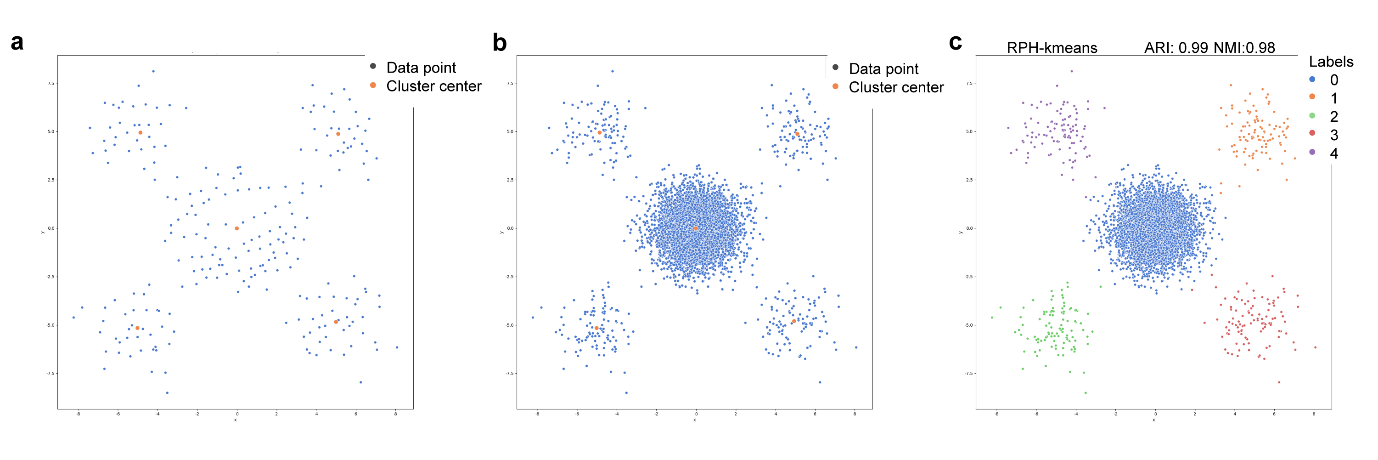


**Figure S3.** Results for simulation 2-D data using RPH-kmeans (default configurations). **a)** Depicts the skeleton points using random projection hashing, and the identified initial cluster centers are shown. **b)** Data points with final cluster center points. **c)** Clustering results are shown, with ARI=0.99 and NMI=0.98.

**Supplementary Note I: Estimation of the number of clusters**

We provide a method to determine the number of clusters $K$ using the weighted skeleton points generated by random projection hashing (Algorithm 2). Inspired by the work of Pelleg (1), we choose the model with the highest Bayesian Information Criterion (BIC) scores. Since the original BIC could not be calculated with weighted data directly, we slightly modified the formula as follows. Let $S=\{\boldsymbol{x}_{i},w_{i}{\}}_{i=1}^{N_{s}}$ be the skeleton data points with dimension $d$, $\{\boldsymbol{c}_{k}{\}}_{k=1}^{K}$ be the cluster centers produced by clustering $S$ with weighted k-means++ (Algorithm S2), $S_{k}\in S$ be the set of points that has $\boldsymbol{c}_{k}$ as their closest centroid, $R=\sum_{i=1}^{N_{s}} w_{i}\mathrm{and}R_{k}=\sum_{\left( \boldsymbol{x}_{i},w_{i} \right)\in S_{k}} w_{i}$. The BIC of a weighted k-means model is defined as:

$$BIC=L\left( S \right)-\frac{p}{2}\log R,$$

where $L\left( S \right)$ is the log-likelihood of weighted data $S$ and $p$ is the number of parameters in the model. Assuming that each cluster is drawn from identical spherical Gaussian distribution with covariance matrix $\sum= \text{diag}\left( \hat{\sigma}^{2} \right)$, the probability of each point is calculated by:

$$P\left( \boldsymbol{x}_{i} \right)=\frac{R_{\left( i \right)}}{R}\cdot\frac{1}{\left( 2\pi\hat{\sigma}^{2} \right)^{\frac{d}{2}}}\exp\left( -\frac{1}{2\hat{\sigma}^{2}}||\boldsymbol{x}_{i}-\boldsymbol{c}_{\left( i \right)}{||}_{2}^{2} \right),$$

where the subscript $\left( i \right)$ refers to the cluster to which $\boldsymbol{x}_{i}$ belongs and $\hat{\sigma}^{2}$ is calculated by:

$$\hat{\sigma}^{2}=\frac{1}{d\left( R-K \right)}\sum_{i=1}^{N_{s}} w_{i}||\boldsymbol{x}_{i}-\boldsymbol{c}_{\left( i \right)}{||}_{2}^{2}.$$

The log-likelihood of all data is:

$$L\left( S \right)=\log\prod_{i=1}^{N_{s}} P\left( \boldsymbol{x}_{i} \right)^{w_{i}}$$

$$=\sum_{i=1}^{N_{s}} w_{i}\left[ \log R_{\left( i \right)}-\log R-\frac{d}{2}\log\left( 2\pi\hat{\sigma}^{2} \right) \right]-\frac{d\left( R-K \right)}{2}$$

$$=\sum_{k=1}^{K} \left[ R_{k}\log R_{k}-R_{k}\log R-\frac{dR_{k}}{2}\log\left( 2\pi\hat{\sigma}^{2} \right)-\frac{d\left( R_{k}-1 \right)}{2} \right]$$

$$=\sum_{k=1}^{K} L\left( S_{k} \right).$$

The number of free parameters $p=K\left( d+1 \right)$ consists of $K$ cluster centers, $K-1$ class probabilities, and one variance estimate. It should be mentioned that there exist some bugs in Pelleg’s description(1), and we derived the formula above based on the corrected version of BIC proposed at <https://github.com/bobhancock/goxmeans/blob/master/doc/BIC_notes.pdf>.

To estimate the optimal cluster number with skeleton data $S$, we calculate a series of BIC score by clustering $S$ with different $k$ and find the knee point of the BIC curve using ‘Kneedle’ (2). The pseudo-code is described in Algorithm S1.

**Algorithm S1: Estimation of the number of clusters**


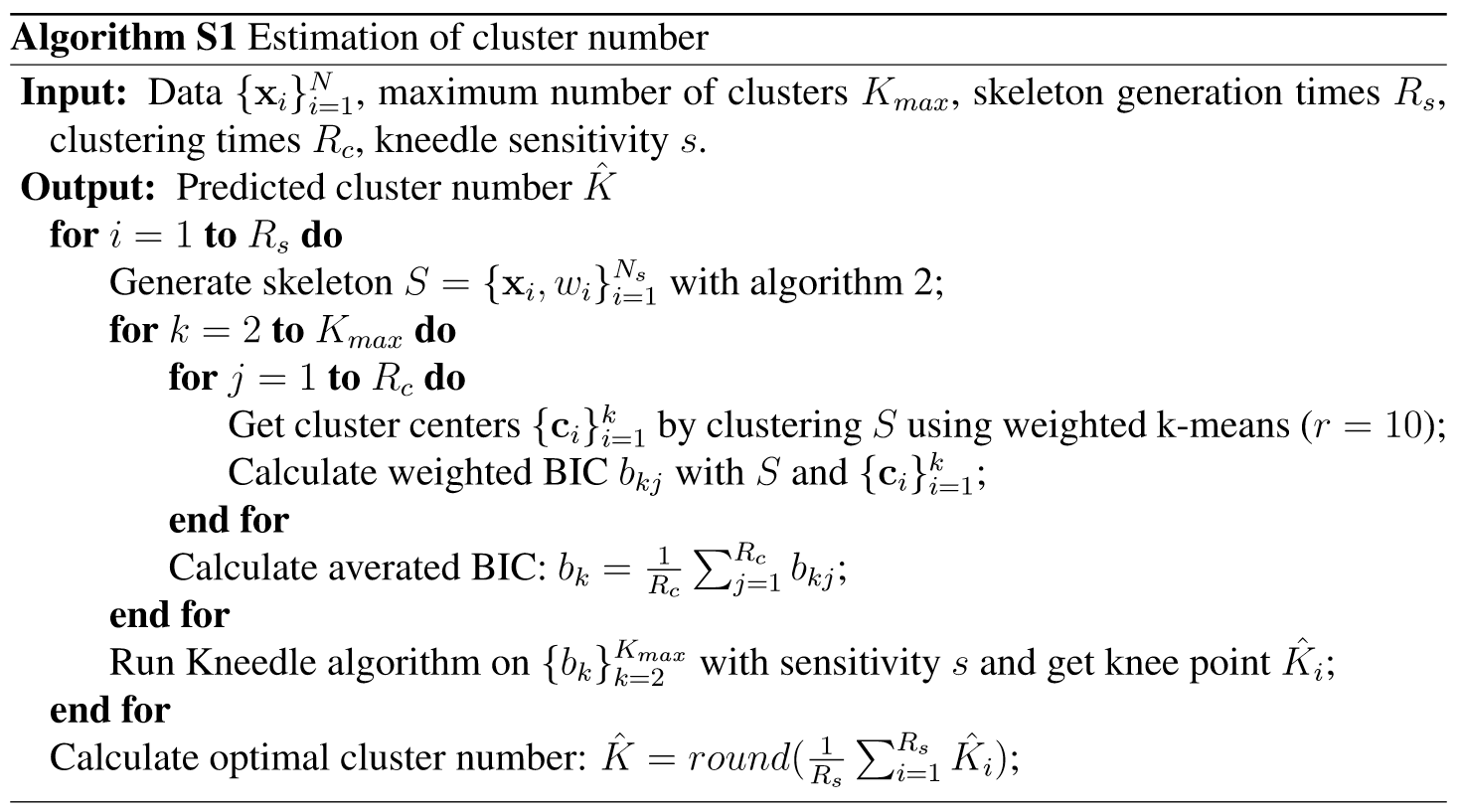


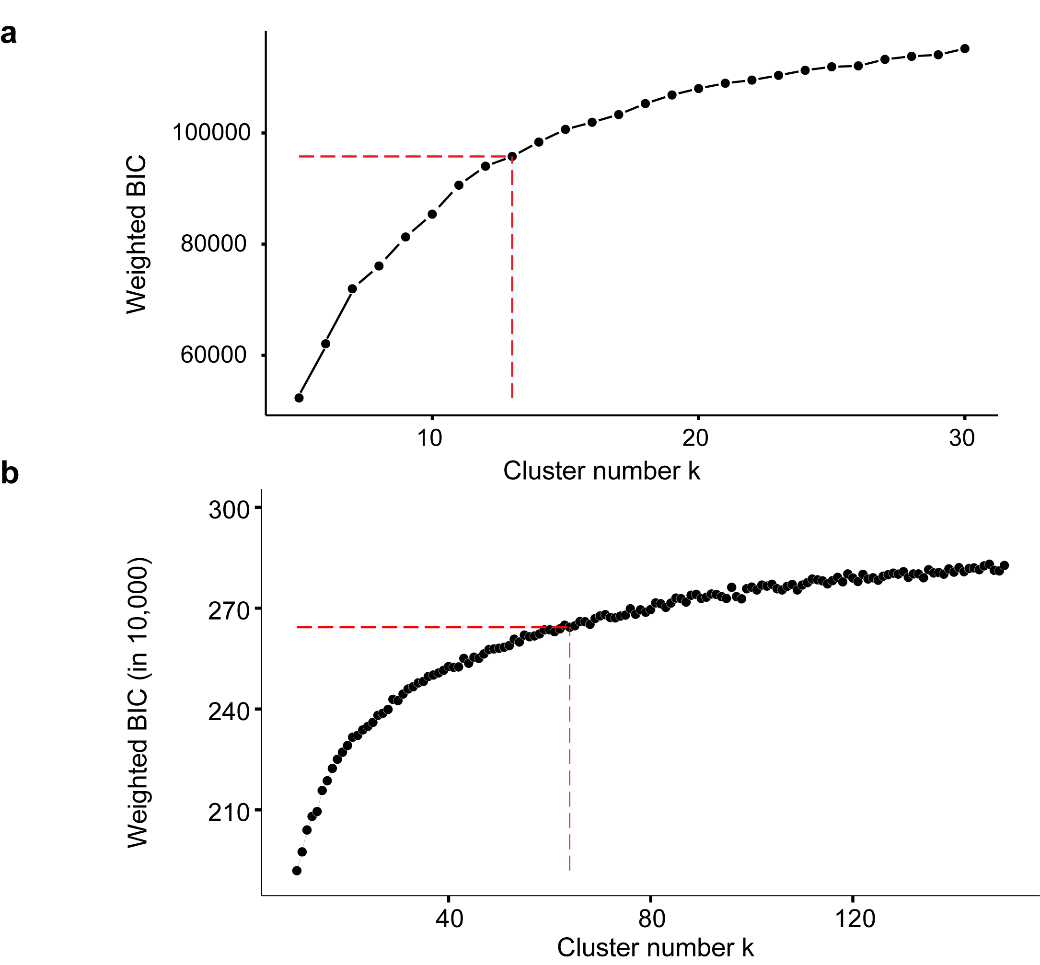


**Figure S4.** Estimation of the number of clusters using weighted BIC. **a)** Results on PBMC 68k dataset, with ‘kneedle’ point determined at k=13. **b)** Results on 1 million neural dataset, with ‘kneedle’ point at k=64.

**Table S1: Estimation of Clusters:**

| **Dataset** | **Given # of  cell types** | **Gene exprs + SC3** | **PCA+ours (s = 3)** | **AIDE+ours (s = 3)** | |
| --- | --- | --- | --- | --- | --- |
| Mouse Brain | 7 |  | 11 | 8 |  |
| PBMC 68k | 10 |  | 9 | 13 |  |
| Mouse Retina | 19 |  | 11 | 9 |  |
| PBMC 4k | 8 | 16 | 8 | 7 |  |
| Mouse Bladder | 16 | 55 | 12 | 10 |  |
| Mouse ES | 4 | 13 | 6 | 6 |  |
| Worm neuron | 10 | 348 | 12 | 10 |  |

**Supplementary algorithms:**

**Algorithm S2: Weighted k-means**


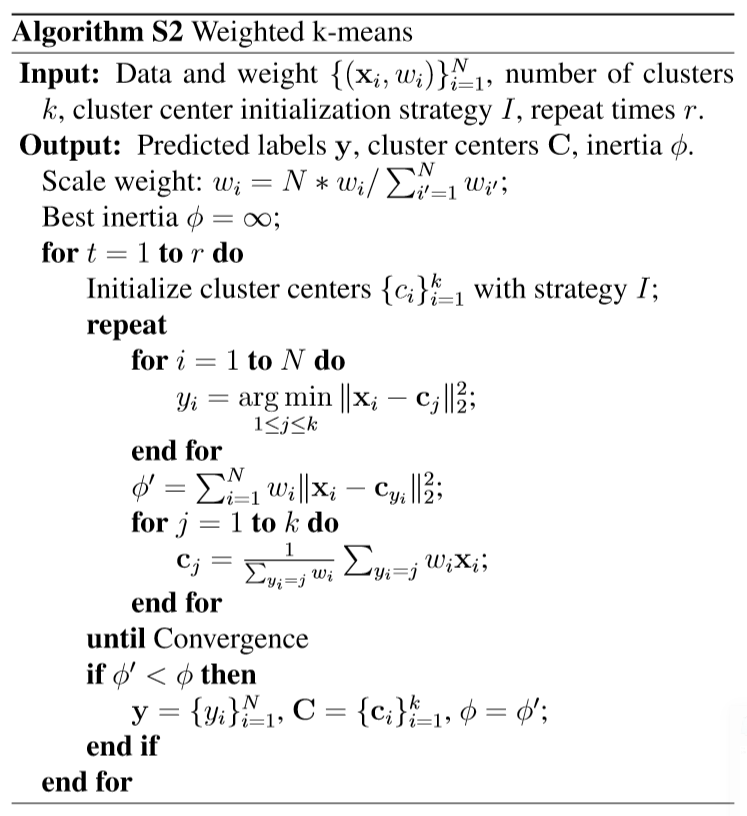


**Algorithm S3-4: Bucket Correction**


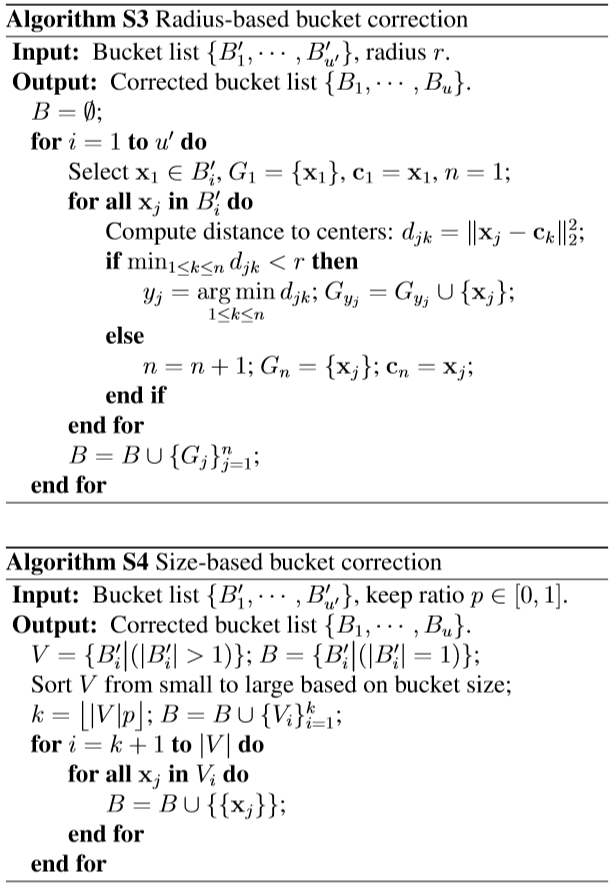


**Effect of hyper-parameters on AIDE:**


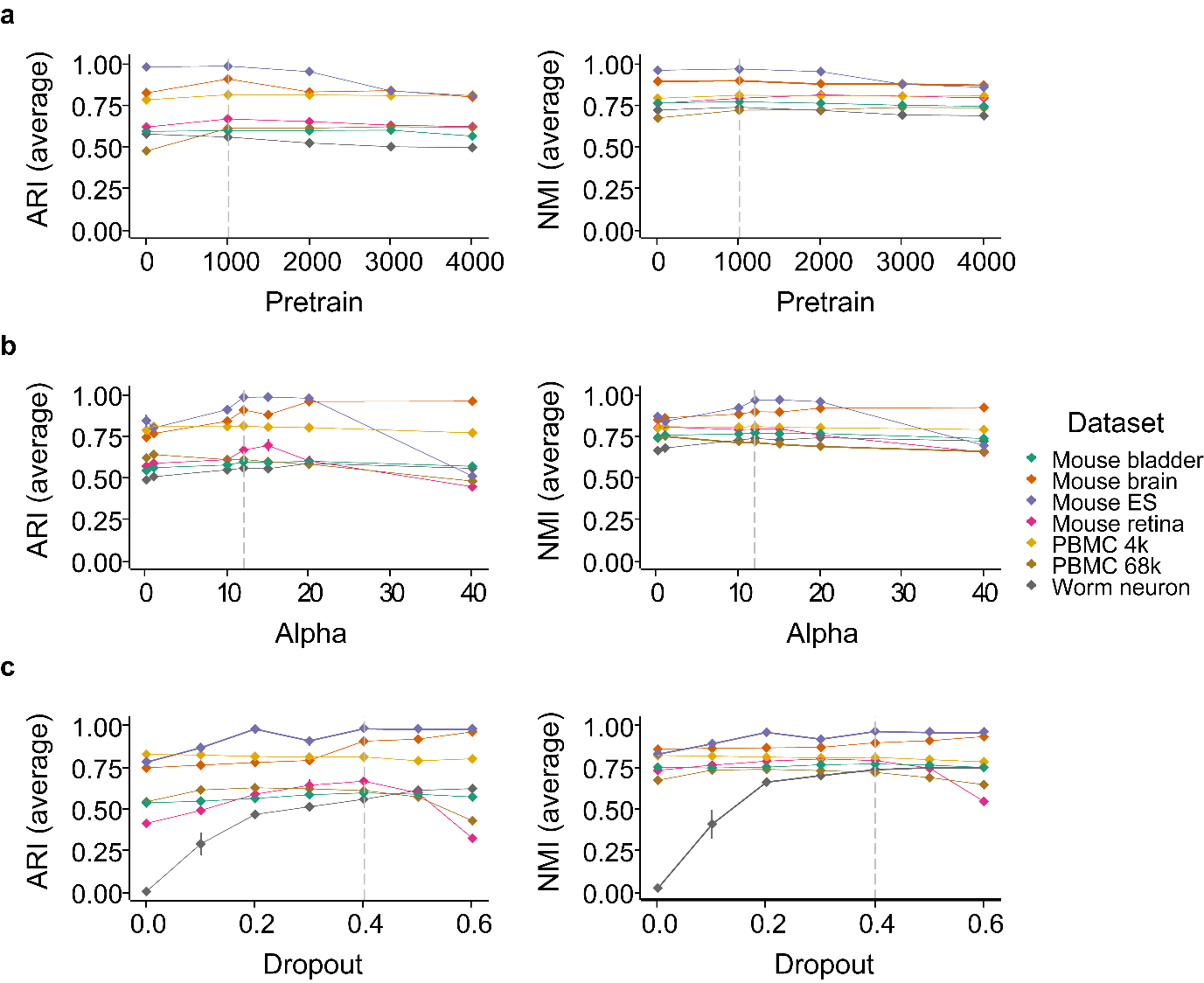


**Figure S5.** Effect of parameters on benchmarked datasets (default parameters: pretrain=1,000, alpha=12, dropout=0.4). **a-c)** The color points mark the average value, and vertical line indicates the 95% confidence interval. We recorded 10 clustering results for 5 embeddings generated using the same parameters (total of 50 observations). The grey dotted line denotes the default parameter value. **a)** depicts the ARI and NMI results using different pretraining steps (0, 1,000, 2,000, 3,000, and 4,000) for each dataset. **b-c)** Similarly for the parameters of alpha (0.1, 1, 10, 12, 20, 40), and dropout (0, 0.1, 0.2, 0.3, 0.4, 0.5, 0.6).

**Hyperparameters:**

**Table S2. Default configurations of AIDE**

| **Hyperparameter** | **Value** |
| --- | --- |
| $\alpha$ * | 12.0 |
| Pretraining steps * | 1,000 |
| Early stop * | True |
| Dropout rate * | 0.4 |
| Learning rate | 0.0001 |
| Batch size | 256 |
| Minimum training steps | 4,000 |
| Maximum training steps | 20,000 |

* indicates that we only changed these parameters in all our experiments on real datasets.

**Table S3. Configuration of AIDE in datasets**

| **Hyperparameter** | **Mouse brain** | **PBMC 68k** | **Mouse retina** | **PBMC 4k** | **Mouse bladder** | **Mouse ES** | **Worm neuron** |  |
| --- | --- | --- | --- | --- | --- | --- | --- | --- |
| $\alpha$ | 40.0 | 1.0 | 20.0 | 1.0 | 40.0 | 15.0 | 20.0 |  |
| Pretraining steps |  | 2,000 | 2,000 | 2,000 | 2,000 |  |  |  |
| Early stop |  |  |  |  | False |  |  |  |
| Dropout rate |  |  |  | 0.2 |  |  | 0.5 |  |

**Note:** For all other datasets mentioned in the experiments of the main paper, we used the default parameters of AIDE.

**Table S4. Default configurations of RPH-kmeans**

| **Hyperparameter** | **Value** |
| --- | --- |
| Quantization step w | Median of ($\vert\vert\boldsymbol{z}_{i}-\boldsymbol{z}_{j}{\vert\vert}_{2}$) * 0.5 |
| Number of LSH functions, l | 5 |
| Maximum number of skeleton points, m | 2,000 |
| Repeat times, $\tau$ | 1 |
| Bucket correction strategy, f | None |

**Table S5. Configurations of RPH-kmeans used for AIDE**

| **Hyperparameter** | **Mouse brain** | **PBMC 68k** | **Mouse retina** | **PBMC 4k** | **Mouse bladder** | **Mouse ES** | **Worm neuron** |  |
| --- | --- | --- | --- | --- | --- | --- | --- | --- |
| Quantization step w |  |  |  |  | 0.5 |  |  |  |
| Number of LSH functions, l |  |  |  |  | 10 |  |  |  |
| Maximum number of skeleton points, m |  | 6,000 |  |  |  |  |  |  |
| Bucket correction strategy, f |  |  |  | Bucket size |  |  | Bucket size |  |
| Parameter of f |  |  |  | 0.5 |  |  | 0.5 |  |

**Note:** For all other datasets mentioned in the experiments of the main paper, we used the default parameters of RPH-kmeans**.**

**Table S6. Configurations of RPH-kmeans used for PCA**

| **Hyperparameter** | **Mouse brain** | **PBMC 68k** | **Mouse retina** | **PBMC 4k** | **Mouse bladder** | **Mouse ES** | **Worm neuron** |  |
| --- | --- | --- | --- | --- | --- | --- | --- | --- |
| Quantization step w |  | 5.5 | 14.0 |  |  |  |  |  |
| Number of LSH functions, l | 7 | 6 |  |  |  |  |  |  |
| Maximum number of skeleton points, m |  | 4,000 |  |  | 500 |  |  |  |
| Bucket correction strategy, f |  |  | Radius | Radius | Bucket size | Radius | Radius |  |
| Parameter of f |  |  | 13.5 | 13.0 | 0.8 | 15.0 | 7.5 |  |

**Note:** This corresponds to the results of Figure S7 (for ‘Mouse brain’ and ‘Mouse retina’ datasets) as well as the raw results of PCA+RPH-kmeans (best) in Supplementary Note IV (Table S22).

**Rare cell type simulations and scalability:**


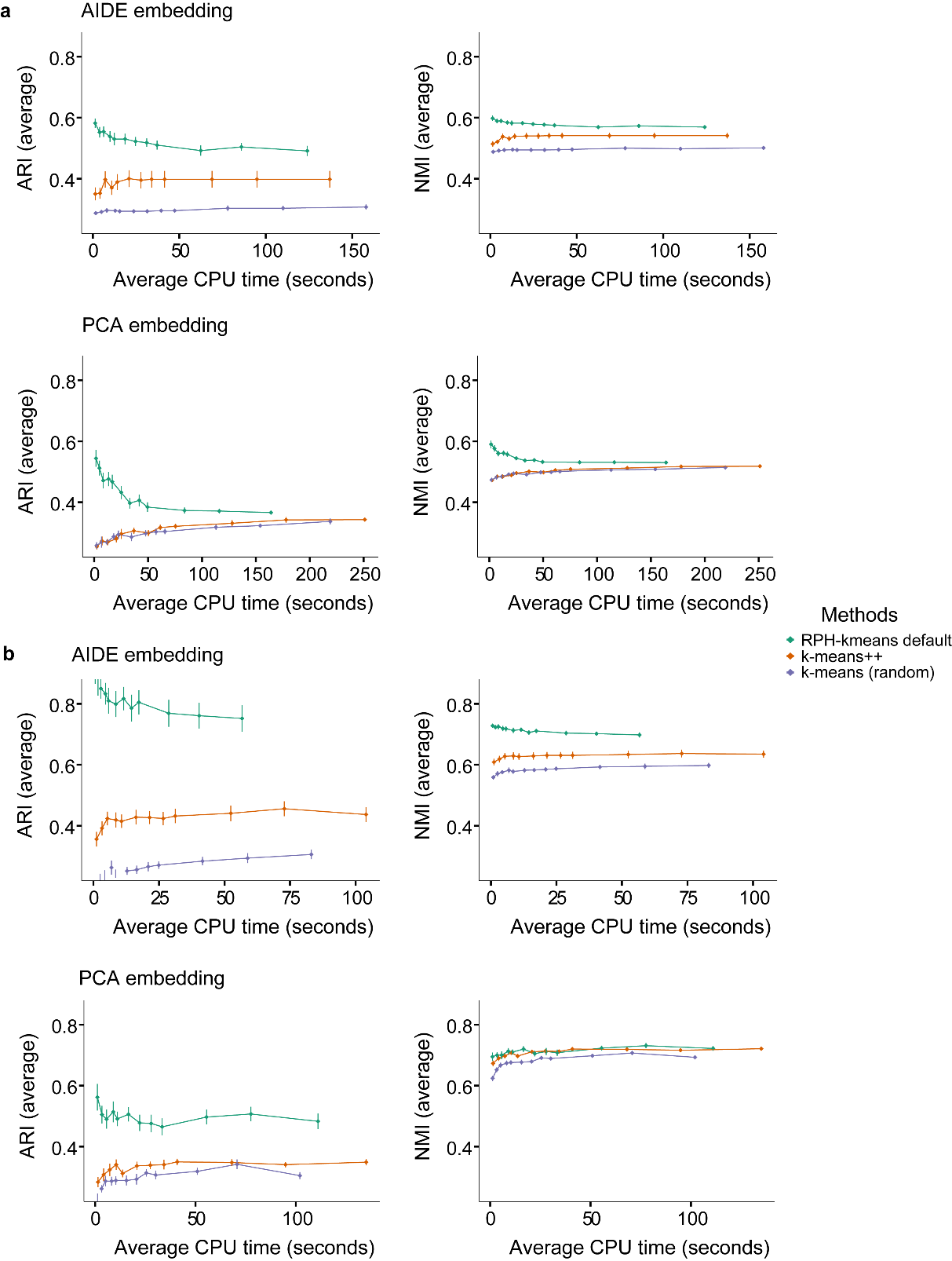


**Figure S6.** Additional simulations on rare cell type detection. **a)** Comparison of conventional k-means initialization algorithms and RPH-kmeans on the ‘PBMC 68k’ dataset; we retained the two largest clusters and sampled 500 cells for each of the remaining cell types. Clustering is applied to both AIDE and PCA embeddings. **b)** Similarly, for the ‘Shekhar retina’ dataset. Vertical lines imply a 95% confidence interval for the recorded results. AIDE and RPH-kmeans were run with default parameters, while PCA, k-means++ and k-means (random initialization) were run with *sklearn* default parameters (PCA was reduced to 256 dimensions). The time was obtained by increasing the number of initializations (termed as repeat times $r$ in Algorithm 1 and S2); we set the number to 1, 3, 5, 6, 10, 15, 20, 25, 30, 50, 70, 100.


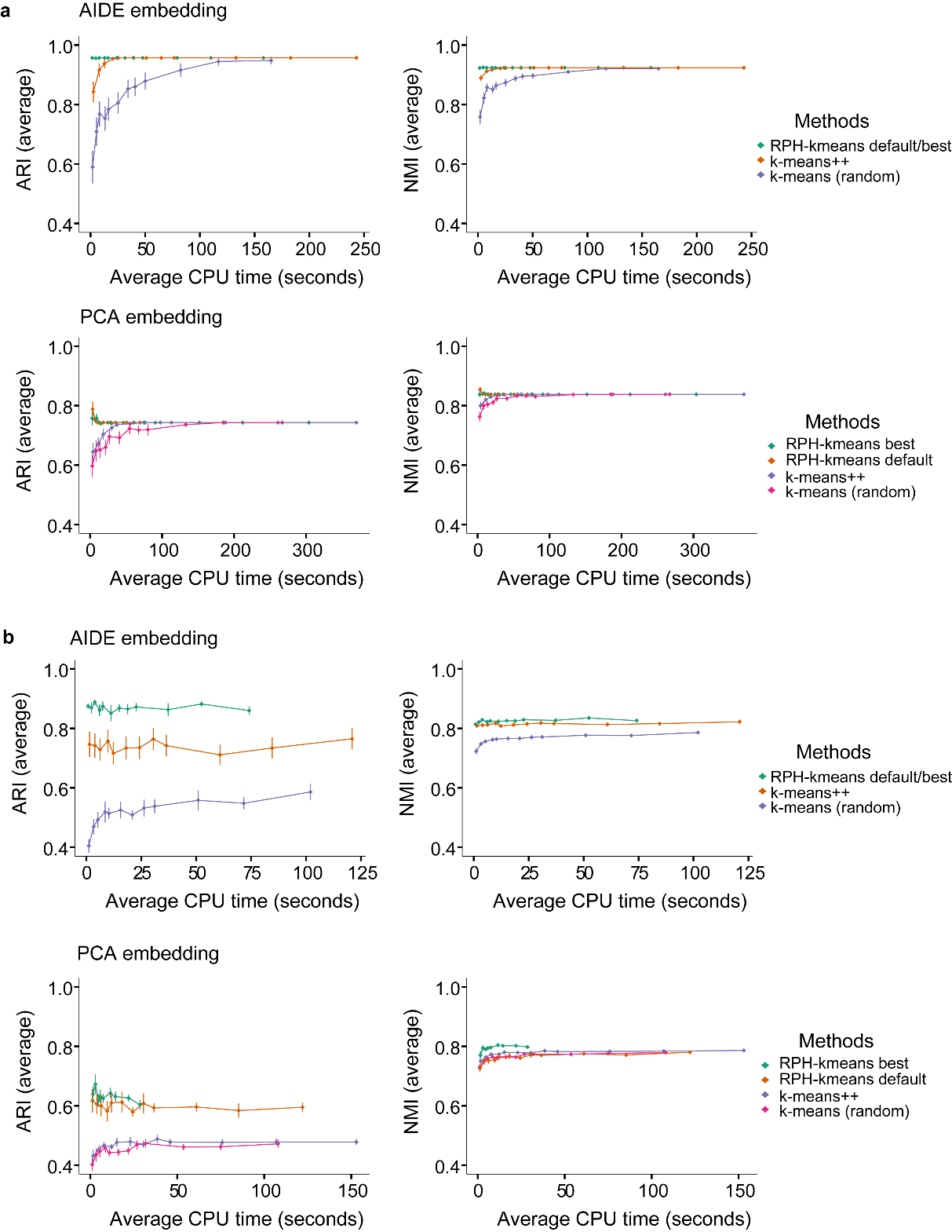


**Figure S7.** Comparing the convergence time of RPH-kmeans and its performance. **a)** Comparison of conventional k-means algorithms and RPH-kmeans on the ‘Mouse brain’ dataset; clustering is applied to both AIDE and PCA embeddings. Both default and tuned RPH-kmeans were compared in PCA embedding. **b)** Similarly for the ‘Mouse retina’ dataset. Vertical lines imply a 95% confidence interval for the recorded results. In general, RPH-kmeans leads to a faster convergence and better results compared to current conventional k-means initialization methods. We used tuned parameters for the AIDE embeddings; both default and tuned paramters for RPH-kmeans; and *sklearn* default parameters for PCA, k-means++ and k-means (random initialization), reducing to 256 PCA dimensions. The time was obtained by increasing the number of initializations (termed as repeat times $r$ in Algorithm 1 and S2); we set the number to 1, 3, 5, 6, 10, 15, 20, 25, 30, 50, 70, 100.

**Supporting evidence for biological analysis:**

**
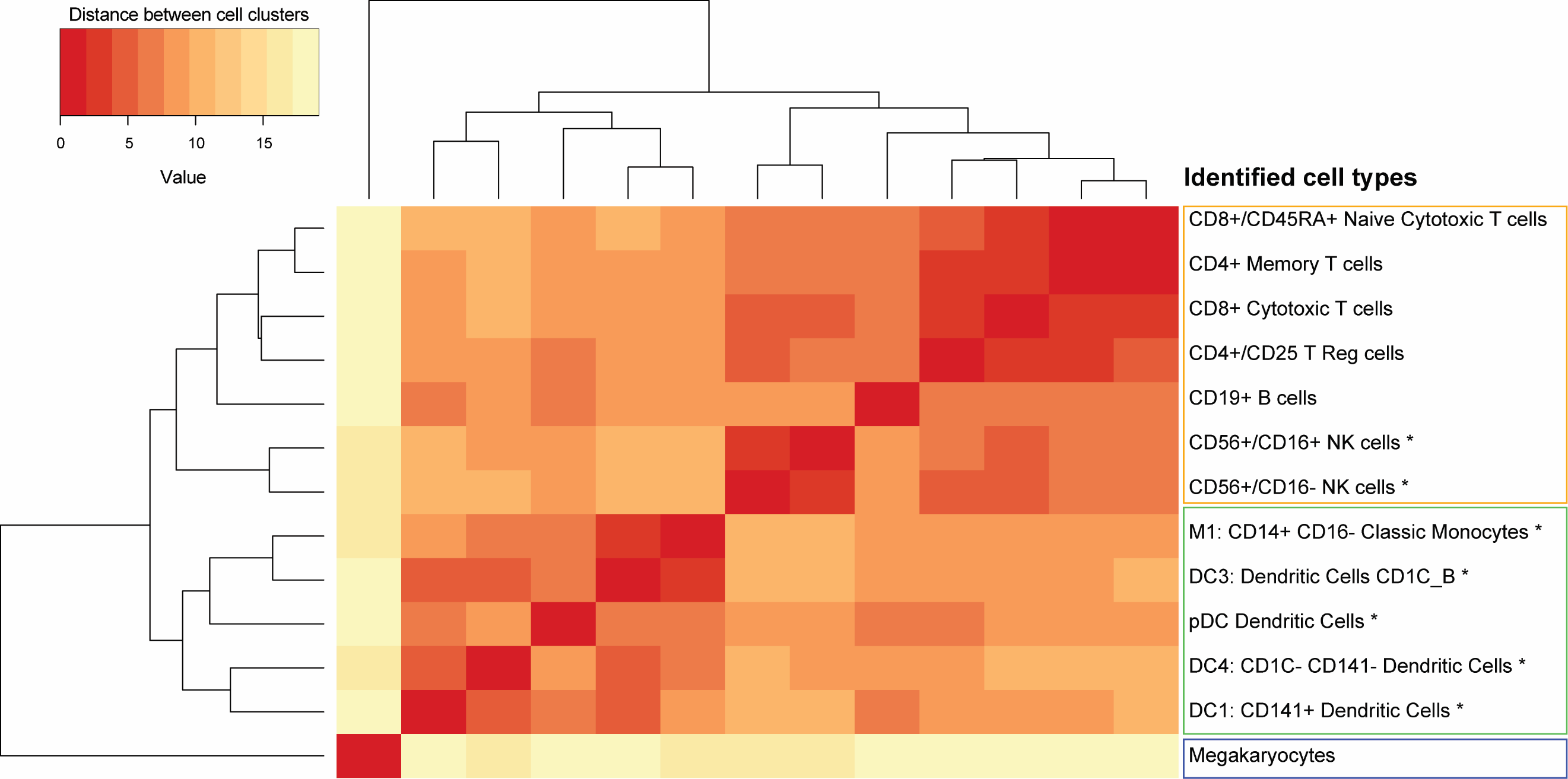
**

**Figure S8. Heatmap visualization of the cell cluster means (PBMC 68k dataset).**

**
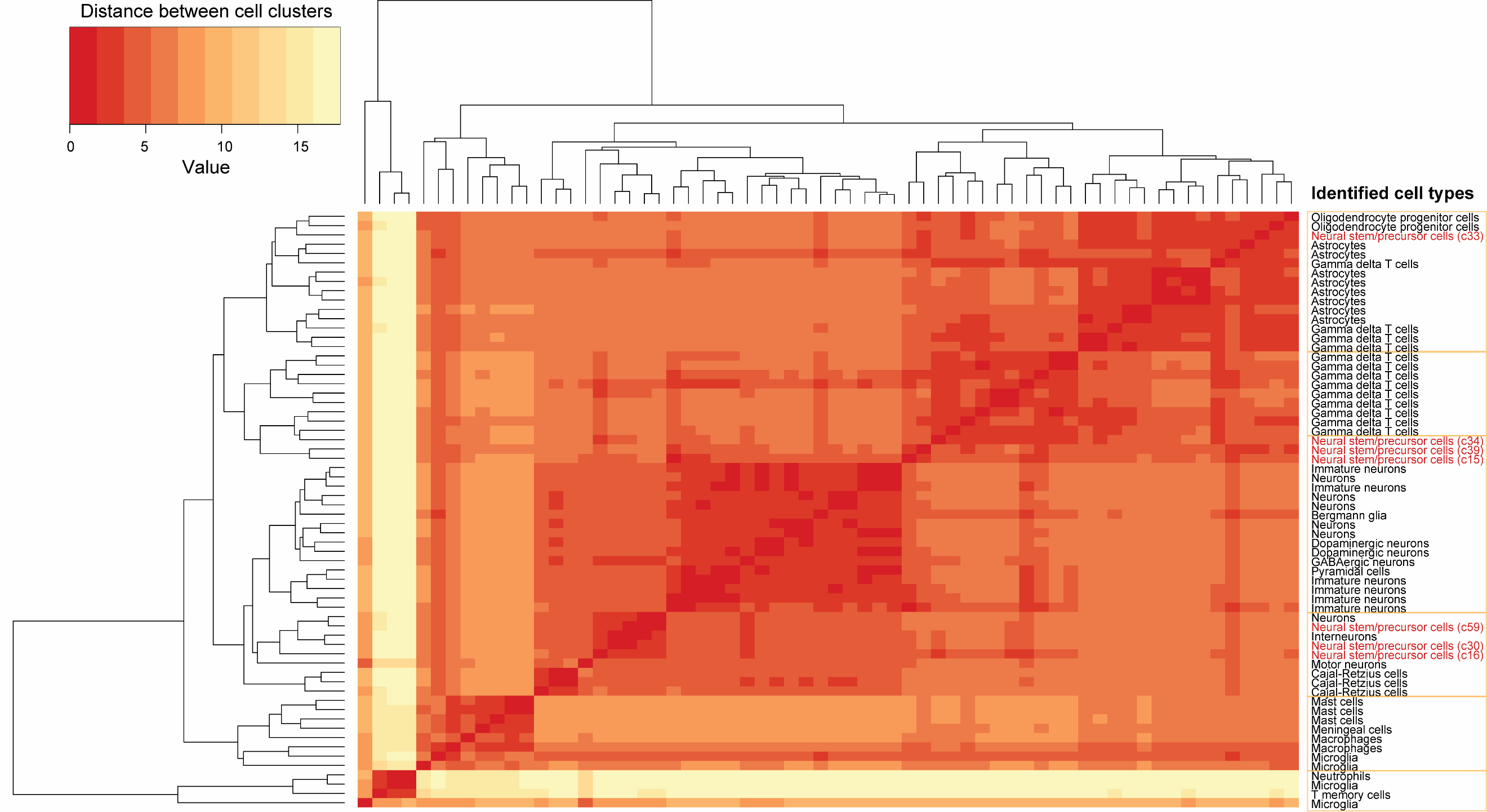
Figure S9. Heatmap visualilzation of the cell cluster means (1.3 neural cell dataset).**

**Table S7: MGH107 markers (Excel file)**

**Table S8: 1m Neural cell type assignment (Excel file)**

**Table S9: Additional markers referenced to identify neural cell types**

| **Cell type** | **Markers** |  |
| --- | --- | --- |
| Bergmann glia | *Hopx, Acsbg1 (3)* | *Non-neurons* |
| Meningeal cells | *Aldh1a2, Vtn (3,4)* |  |
| Microglia | *Csf1r (5)* |  |
| Astrocytes | *Gfap, Tbr1 (6)* |  |
| Oligodendrocyte progenitor cells | *Olig1, Olig2 (7,8)* |  |
| Neural stem/precursor cells | *Egfr, Stmn1, Aldoc* (6,9) | *Neuron cells* |
| Immature neurons | *Tbr1, Neurod1 (10)* |  |
| Neurons | *Meg3, Slc12a5, Eno2 (11)* |  |
| Dopaminergic neurons | *Neurod6 (12)* |  |
| Interneurons | *Camk2a, Pacsin1, Abr (3,4,13)* |  |
| Motor neurons | *Sim1 (14)* |  |
| GABAergic neurons | *Gad1, Gad2, Slc32a1 (3,15)* |  |
| Cajal-Retzius cells | *Reln, Prtn3 (16)* |  |
| Pyramidal cells | *Pcp4 (17)* |  |
| Mast cells | *Lamp-1, Lamp-2 (18)* | *Immune cells* |
| Megakaryocytes | *Gata1 (19)* |  |
| Macrophages | *Csf1r, Tyrobp, Lyz2 (20-22)* |  |
| Neutrophils | *Car1, Clec4e (23)* |  |
| Gamma delta T cells | *Ccnb1, Top2a, H2afz, Hmgb2 (24)* |  |

**Table S10: Markers used for identifying neural stem/precursor cell subpopulations**

| **Cell type** | **Markers** |
| --- | --- |
| Quiescent neural stem cell (qNSC) markers | *Clu, Id3 (6,9)* |
| Activated neural stem cell (aNSC) markers | *Egfr, Atp1a2, Gfap, Prom1 (6,9)* |
| Neurogenesis markers | *Dlx1, Dlx2, Dcx, Dlx6as1 (6)* |
| Astrocytic markers | *Fgfr3, Gja1, Jag1 (6)* |

**Table S11: Annotation of neural stem/precursor cell subpopulations**

| **Cluster** | **Cell type** | **Markers** |
| --- | --- | --- |
| 30 | aNSC/NPC for neurons | *Neurogenesis (Dlx1, Dlx2), Meg3** |
| 59 |  | *Neurogenesis (Dlx1, Dlx2, Dcx), Meg3** |
| 16 |  | *Neurogenesis (Dlx1, Dlx2)* |
| 15 | Mid- to late-aNSC for neurons | *Ccnd2 (cell-cycling gene), Slc1a3 (GLAST)* |
| 39 | Mid- to late-aNSC for neurons | *aNSC (all), Astrocytic (Gja1), Gfap** |
| 34 | Late-aNSC/NPC for OPCs | *Neurogenesis (Dlx1, Dlx2), Pdgfra*, Olig2** |
| 33 | Early- to mid-aNSC/NPC for OPCs | *Clu, Astrocytic (all), aNSC (all), Gfap** |

* Indicates that the specific marker is unique to the particular cluster


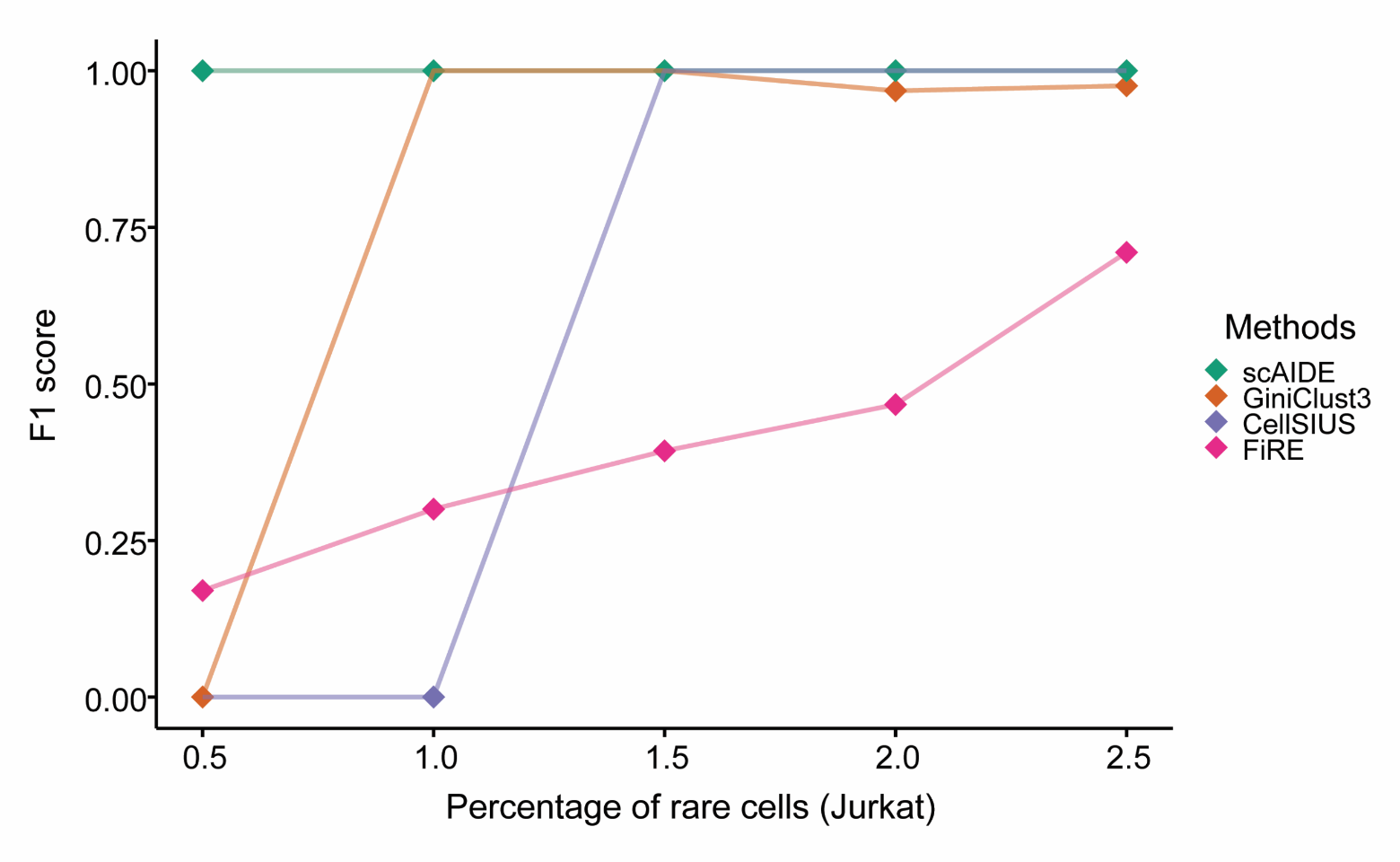


**Figure S10. Rare cell type simulation experiment with Jurkat cells.** F1-score evaluation on simulated rare cell type detection. Each method is performed once, as results were consistent.

We tuned the neighborhood parameter for GiniClust3 (set to 10) to ensure better discovery of rare cell types. The default parameters were used for CellSIUS, FiRE, and scAIDE. For the RPH-kmeans component, we reduced the maximum number of merged points to 20 (max_point=20) as the default value (max_point=2000) exceeds the number of cells. We simply set k=2 for 1%, 1.5%, and 2% for scAIDE and we used an estimated number of clusters (k=3) for 0.5% mixture.

F1 score was calculated with respect to the rare population (Jurkat cells). In the case of three or more clusters, we considered the group with the most identified rare cell type as the primary cluster and combined the rest to one abundant cluster to calculate an F1 score; if one of the cluster assignments matches exactly with the rare cell type, F1 score is considered to be 1.0.

GiniClust3 returned three cluster assignments for 1% (1528, **16**, 12), 1.5% (1528, **24**, 12), and 2% (1526, **34**, 12) and 2.5% (1526, **42**, 12) mixture, as well as scAIDE for 0.5% (**8**, 1527, 13) mixture. F1 score is 1 for all the mentioned cases except for GiniClust3 at 2% and 2.5% mixtures. At 2% mixture, 2 false positives were detected by GiniClust3 with respect to Jurkat cells (cluster of 34 cells with 32 True Positives), and we calculated the F-score by merging the other two clusters (1,526 cells and 12 cells). Similarly, for GiniClust3 at 2.5% mixture. CellSIUS could not detect any subclusters when the mixture was below 1%.


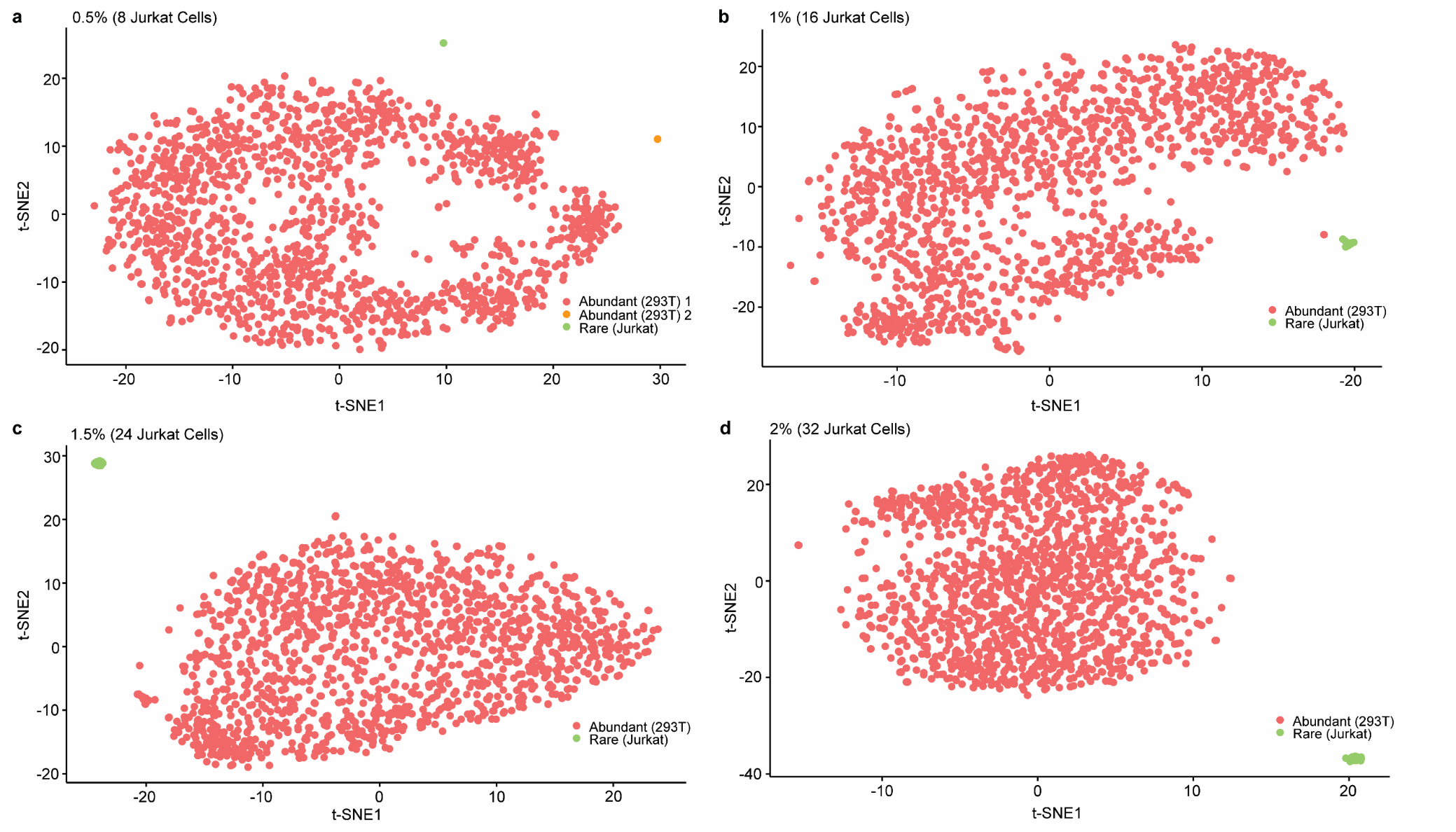


**Figure S11.** **Visualization on AIDE embeddings. a**-**d**). t-SNE visualization on AIDE embeddings for each proportion of rare cell type (Jurkat cells) for 0.5%, 1%, 1.5% and 2% mixtures. The colored labels are predicted labels by scAIDE.

**Clustering analysis on the Mouse ES dataset:**


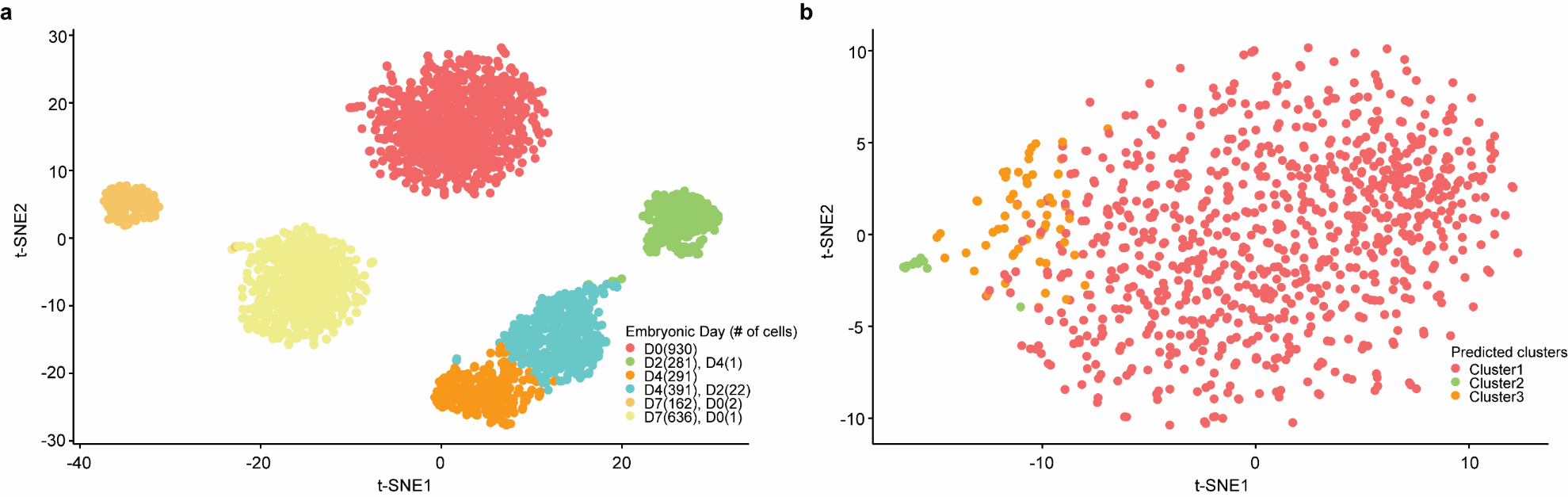


**Figure S12. Visualization of clustering results on Mouse ES dataset. a**). Global clustering reveals six different clusters using scAIDE. The color labels include the distribution of the number of cells in each respective LIF withdrawal time interval. **b**). AIDE embedding on mESC at time D0 (933 cells in total).

By global clustering, scAIDE (default parameters) estimated 6 clusters in the Mouse ES dataset with four withdrawal times (Figure S12a). Two main subpopulations were identified at times D4 and D7, respectively. We cross-referenced with the markers reported in the original publication (25), and identified one subpopulation of D7 cells (in yellow) expressing markers of high pluripotency and primitive endoderm.

To resemble the embryonic stem cell (mESC) analysis in the original publication, we attempted to cluster the mESCs at Day 0 (Figure S12b). We were able to identify a distinct subpopulation (Cluster 2: 16/933 cells), which is *Prdm1*-high. Marker genes (*Prdm1*, *Baat*, *Nsun6*, *Parp4*, *Srgn*, and *Ssh1*) were detected with p-value < 0.01 and log-fold change > 1.5. This matches an identified small cluster of 13 cells from the original publication (25).

**References:**

1. Pelleg, D. and Moore, A.W. (2000) X-means: Extending K-means with Efficient Estimation of the Number of Clusters.*Proceedings of the Seventeenth International Conference on Machine Learning*. Morgan Kaufmann Publishers Inc., pp. 727–734.

2. Satopaa, V., Albrecht, J., Irwin, D. and Raghavan, B. (2011) Finding a “Kneedle” in a Haystack: Detecting Knee Points in System Behavior. *Proceedings of the 2011 31st International Conference on Distributed Computing Systems Workshops*. IEEE Computer Society, pp. 166–171.

3. Zeisel, A., Hochgerner, H., Lonnerberg, P., Johnsson, A., Memic, F., van der Zwan, J., Haring, M., Braun, E., Borm, L.E., La Manno, G. *et al.* (2018) Molecular Architecture of the Mouse Nervous System. *Cell*, **174**, 999-1014 e1022.

4. Tepe, B., Hill, M.C., Pekarek, B.T., Hunt, P.J., Martin, T.J., Martin, J.F. and Arenkiel, B.R. (2018) Single-Cell RNA-Seq of Mouse Olfactory Bulb Reveals Cellular Heterogeneity and Activity-Dependent Molecular Census of Adult-Born Neurons. *Cell Rep*, **25**, 2689-2703 e2683.

5. Gerber, Y.N., Saint-Martin, G.P., Bringuier, C.M., Bartolami, S., Goze-Bac, C., Noristani, H.N. and Perrin, F.E. (2018) CSF1R Inhibition Reduces Microglia Proliferation, Promotes Tissue Preservation and Improves Motor Recovery After Spinal Cord Injury. *Front Cell Neurosci*, **12**, 368.

6. Dulken, B.W., Leeman, D.S., Boutet, S.C., Hebestreit, K. and Brunet, A. (2017) Single-Cell Transcriptomic Analysis Defines Heterogeneity and Transcriptional Dynamics in the Adult Neural Stem Cell Lineage. *Cell Rep*, **18**, 777-790.

7. Zhou, Q. and Anderson, D.J. (2002) The bHLH transcription factors OLIG2 and OLIG1 couple neuronal and glial subtype specification. *Cell*, **109**, 61-73.

8. Lu, Q.R., Sun, T., Zhu, Z., Ma, N., Garcia, M., Stiles, C.D. and Rowitch, D.H. (2002) Common developmental requirement for Olig function indicates a motor neuron/oligodendrocyte connection. *Cell*, **109**, 75-86.

9. Xie, K., Liu, Z., Chen, N. and Chen, T. (2020) redPATH: Reconstructing the Pseudo Development Time of Cell Lineages in Single-Cell RNA-Seq Data and Applications in Cancer. 2020.2003.2005.977686.

10. Duan, L., Zhang, X.-D., Miao, W.-Y., Sun, Y.-J., Xiong, G., Wu, Q., Li, G., Yang, P., Yu, H. and Li, H.J.N. (2018) PDGFRβ cells rapidly relay inflammatory signal from the circulatory system to neurons via chemokine CCL2. **100**, 183-200. e188.

11. Rosenberg, A.B., Roco, C.M., Muscat, R.A., Kuchina, A., Sample, P., Yao, Z., Graybuck, L.T., Peeler, D.J., Mukherjee, S., Chen, W. *et al.* (2018) Single-cell profiling of the developing mouse brain and spinal cord with split-pool barcoding. *Science*, **360**, 176-182.

12. Kramer, D.J., Risso, D., Kosillo, P., Ngai, J. and Bateup, H.S. (2018) Combinatorial Expression of Grp and Neurod6 Defines Dopamine Neuron Populations with Distinct Projection Patterns and Disease Vulnerability. *eNeuro*, **5**.

13. Kalish, B.T., Cheadle, L., Hrvatin, S., Nagy, M.A., Rivera, S., Crow, M., Gillis, J., Kirchner, R. and Greenberg, M.E. (2018) Single-cell transcriptomics of the developing lateral geniculate nucleus reveals insights into circuit assembly and refinement. *Proc Natl Acad Sci U S A*, **115**, E1051-E1060.

14. Yang, X., Tomita, T., Wines-Samuelson, M., Beglopoulos, V., Tansey, M.G., Kopan, R. and Shen, J. (2006) Notch1 signaling influences v2 interneuron and motor neuron development in the spinal cord. *Dev Neurosci*, **28**, 102-117.

15. Chowdhury, S., Matsubara, T., Miyazaki, T., Ono, D., Fukatsu, N., Abe, M., Sakimura, K., Sudo, Y. and Yamanaka, A. (2019) GABA neurons in the ventral tegmental area regulate non-rapid eye movement sleep in mice. *Elife*, **8**.

16. Griveau, A., Borello, U., Causeret, F., Tissir, F., Boggetto, N., Karaz, S. and Pierani, A. (2010) A novel role for Dbx1-derived Cajal-Retzius cells in early regionalization of the cerebral cortical neuroepithelium. *PLoS Biol*, **8**, e1000440.

17. San Antonio, A., Liban, K., Ikrar, T., Tsyganovskiy, E. and Xu, X. (2014) Distinct physiological and developmental properties of hippocampal CA2 subfield revealed by using anti-Purkinje cell protein 4 (PCP4) immunostaining. *J Comp Neurol*, **522**, 1333-1354.

18. Grutzkau, A., Smorodchenko, A., Lippert, U., Kirchhof, L., Artuc, M. and Henz, B.M. (2004) LAMP-1 and LAMP-2, but not LAMP-3, are reliable markers for activation-induced secretion of human mast cells. *Cytometry A*, **61**, 62-68.

19. Kuhl, C., Atzberger, A., Iborra, F., Nieswandt, B., Porcher, C. and Vyas, P. (2005) GATA1-mediated megakaryocyte differentiation and growth control can be uncoupled and mapped to different domains in GATA1. *Mol Cell Biol*, **25**, 8592-8606.

20. Yahara, Y., Barrientos, T., Tang, Y.J., Puviindran, V., Nadesan, P., Zhang, H., Gibson, J.R., Gregory, S.G., Diao, Y., Xiang, Y. *et al.* (2020) Erythromyeloid progenitors give rise to a population of osteoclasts that contribute to bone homeostasis and repair. *Nat Cell Biol*, **22**, 49-59.

21. Murray, P.J. and Wynn, T.A. (2011) Obstacles and opportunities for understanding macrophage polarization. *J Leukoc Biol*, **89**, 557-563.

22. Helming, L., Tomasello, E., Kyriakides, T.R., Martinez, F.O., Takai, T., Gordon, S. and Vivier, E. (2008) Essential role of DAP12 signaling in macrophage programming into a fusion-competent state. *Sci Signal*, **1**, ra11.

23. Giladi, A., Paul, F., Herzog, Y., Lubling, Y., Weiner, A., Yofe, I., Jaitin, D., Cabezas-Wallscheid, N., Dress, R., Ginhoux, F. *et al.* (2018) Single-cell characterization of haematopoietic progenitors and their trajectories in homeostasis and perturbed haematopoiesis. *Nature Cell Biology*, **20**, 836-846.

24. Franzen, O., Gan, L.M. and Bjorkegren, J.L.M. (2019) PanglaoDB: a web server for exploration of mouse and human single-cell RNA sequencing data. *Database (Oxford)*, **2019**.

25. Klein, A.M., Mazutis, L., Akartuna, I., Tallapragada, N., Veres, A., Li, V., Peshkin, L., Weitz, D.A. and Kirschner, M.W. (2015) Droplet barcoding for single-cell transcriptomics applied to embryonic stem cells. *Cell*, **161**, 1187-1201.
