# Supplementary material for: scAIDE: clustering of large-scale single-cell RNA-seq data reveals putative and rare cell types
Source: NAR Genom Bioinform. 2020 Oct 9;2(4):lqaa082. doi: 10.1093/nargab/lqaa082 (PMC7671411; doi:10.1093/nargab/lqaa082)
Supplement: lqaa082_Supplemental_Files [file lqaa082_supplemental_files.zip › Supplementary Notes II-VII.pdf]

# Supplementary Note II

## Comparison of different components of AIDE

In order to show the performance of the novel architecture, AIDE, we compared the clustering performance of AIDE to individual components (AutoEncoder, DeepMDS). We simply applied the k-means++ clustering algorithm to the embeddings generated from AIDE, AutoEncoder, and DeepMDS. The number of initializations (n_init/repeat times $r$) was set to 10.

For the AutoEncoder component, we used the smallest hidden vectors (256-dim) as input to perform clustering. As for DeepMDS (embedding without imputation), we minimized the following loss function to generate a low-dimensional representation $\mathbf{z}$:

$$L = \alpha \frac{1}{|D_p|} \sum_{(\mathbf{x}_i, \mathbf{x}_\mathbf{j}) \in D_p} \Big| ||\mathbf{z}_i - \mathbf{z}_j||_2^2 - \varphi(\mathbf{x}_i, \mathbf{x}_j)^2 \Big|.$$

One main difference between DeepMDS and AIDE is that DeepMDS trains on the distance between original gene expression vectors, $\varphi(\mathbf{x}_i, \mathbf{x}_j)^2$, instead of the imputed gene vectors from AE, $\varphi(\hat{\mathbf{x}}_i, \hat{\mathbf{x}}_j)^2$.

From Table S12-13, we observed that AIDE outperforms individual components in all of the benchmarked datasets.

**Table S12. Performance of AutoEncoder**

| Methods | Mouse brain | | PBMC 68k | | Mouse retina | | PBMC 4k | | Mouse bladder | | Mouse ES | | Worm neuron | |
|---|---|---|---|---|---|---|---|---|---|---|---|---|---|---|
| | ARI | NMI | ARI | NMI | ARI | NMI | ARI | NMI | ARI | NMI | ARI | NMI | ARI | NMI |
| **AIDE (default)** | **0.903 (0.070)** | **0.893 (0.012)** | **0.606 (0.009)** | **0.715 (0.010)** | **0.663 (0.107)** | **0.787 (0.020)** | **0.808 (0.003)** | **0.805 (0.003)** | **0.592 (0.031)** | **0.766 (0.010)** | **0.980 (0.003)** | **0.963 (0.007)** | **0.554 (0.038)** | **0.733 (0.013)** |
| **Dropout-0.4; early stop** | 0.470 (0.033) | 0.683 (0.017) | 0.498 (0.033) | 0.651 (0.010) | 0.564 (0.056) | 0.688 (0.017) | 0.305 (0.034) | 0.472 (0.028) | 0.271 (0.011) | 0.522 (0.009) | 0.314 (0.013) | 0.398 (0.020) | 0.176 (0.024) | 0.357 (0.023) |
| **Dropout-0.4; step 20000** | 0.433 (0.039) | 0.669 (0.028) | 0.521 (0.033) | 0.655 (0.013) | 0.551 (0.097) | 0.669 (0.060) | 0.326 (0.024) | 0.491 (0.019) | 0.266 (0.013) | 0.518 (0.013) | 0.316 (0.022) | 0.401 (0.021) | 0.204 (0.017) | 0.365 (0.022) |
| **Dropout-0.0; early stop** | 0.567 (0.059) | 0.778 (0.035) | 0.550 (0.025) | 0.697 (0.011) | 0.426 (0.052) | 0.753 (0.020) | 0.695 (0.066) | 0.747 (0.023) | 0.468 (0.036) | 0.696 (0.013) | 0.518 (0.036) | 0.667 (0.029) | 0.497 (0.053) | 0.657 (0.023) |
| **Dropout-0.0; step 20000** | 0.592 (0.065) | 0.785 (0.030) | 0.567 (0.022) | 0.701 (0.011) | 0.428 (0.046) | 0.753 (0.018) | 0.674 (0.071) | 0.740 (0.025) | 0.484 (0.036) | 0.701 (0.011) | 0.551 (0.009) | 0.694 (0.012) | 0.515 (0.065) | 0.661 (0.031) |

Note: For AIDE (default), we set the dropout rate to 0.4 and used early stop strategy.

**Table S13. Performance of DeepMDS**

| Methods | Mouse brain | | PBMC 68k | | Mouse retina | | PBMC 4k | | Mouse bladder | | Mouse ES | | Worm neuron | |
|---|---|---|---|---|---|---|---|---|---|---|---|---|---|---|
| | ARI | NMI | ARI | NMI | ARI | NMI | ARI | NMI | ARI | NMI | ARI | NMI | ARI | NMI |
| **AIDE (default)** | **0.903 (0.070)** | **0.893 (0.012)** | **0.606 (0.009)** | **0.715 (0.010)** | **0.663 (0.107)** | **0.787 (0.020)** | **0.808 (0.003)** | **0.805 (0.003)** | **0.592 (0.031)** | **0.766 (0.010)** | **0.980 (0.003)** | **0.963 (0.007)** | **0.554 (0.038)** | **0.733 (0.013)** |
| **Alpha-1.0; early stop** | 0.735 (0.099) | 0.795 (0.0348) | 0.532 (0.0509) | 0.687 (0.0224) | 0.378 (0.0328) | 0.734 (0.0159) | 0.802 (0.0174) | 0.803 (0.00875) | 0.505 (0.0331) | 0.739 (0.0111) | 0.79 (0.0319) | 0.837 (0.0161) | 0.493 (0.0898) | 0.687 (0.0328) |
| **Alpha-1.0; step 20000** | 0.752 (0.0831) | 0.806 (0.0364) | 0.568 (0.0292) | 0.707 (0.0121) | 0.394 (0.05) | 0.744 (0.0189) | 0.764 (0.0552) | 0.794 (0.0201) | 0.528 (0.0427) | 0.744 (0.0143) | 0.805 (0.034) | 0.846 (0.0169) | 0.401 (0.0702) | 0.629 (0.0407) |
| **Alpha-12.0; early stop** | 0.728 (0.0977) | 0.792 (0.0366) | 0.537 (0.0337) | 0.693 (0.0148) | 0.386 (0.0436) | 0.74 (0.019) | 0.785 (0.0488) | 0.798 (0.0203) | 0.486 (0.0311) | 0.73 (0.0123) | 0.791 (0.0608) | 0.841 (0.0364) | 0.494 (0.0773) | 0.679 (0.0356) |
| **Alpha-12.0; step 20000** | 0.774 (0.0994) | 0.812 (0.0479) | 0.573 (0.0463) | 0.711 (0.0188) | 0.387 (0.049) | 0.738 (0.0187) | 0.768 (0.0539) | 0.795 (0.0214) | 0.516 (0.0451) | 0.74 (0.0161) | 0.809 (0.00241) | 0.848 (0.00379) | 0.403 (0.0721) | 0.625 (0.0417) |

Note: For AIDE (default), we set the alpha to 12.0 and used early stop strategy.

## Supplementary Note III

## Comparisons with current state-of-the-art methods

In this section, we included the raw ARI and NMI evaluations for each of our clustering benchmarks (Table S14). We also include the raw results for different parameters used for other methods (Table S15-21). In each of the tables, an average ARI/NMI value is given with its standard deviation in brackets. Each row in the table represents the method with different parameter settings, and the column represents the benchmarked dataset.

We used scDeepCluster, DCA, scVI, scScope, SIMLR, MAGIC, ZIFA, SC3, PCA, and MDS as baselines. All the raw results for each respective method are shown here in Table S14, the best results were plotted in Figure 1B.

scDeepCluster is a model-based deep clustering method that incorporates the ZINB model into their loss function. The output embedding dimension was set to 32-dim (default: 256-64-32) and 256-dim (1024-512-256) in our evaluation. As DCA is designed to denoise single-cell data, so we first denoise the raw data with an output of 32-dim embedding (default: 64-32-64) and 256-dim (1024-512-256-512-1024), followed by normalization and log transformation. Subsequently, we applied k-means++ clustering to the embedding space, the imputed data, and PCA reduced space (to 256-dim). scVI is a deep learning framework that learns a probabilistic representation of single-cell data. We generated embedding of size 10 (default: 128-10) and 256 (512-512-256), and applied k-means++ to the embedding space. scScope is a recurrent neural network model that incorporates batch correction. As the original publication uses only top 1,000 genes as input, we tested its performance for both top 1,000 selected genes and minimally processed (up to 20,000 genes) expression. We first reduced the dimensionality to 50 (with default parameters) and 256 (1024-512-256), then we applied the k-means++ algorithm or phenograph to cluster the data. We used the default parameters for SIMLR, a multi-kernel learning tool, with and without cell normalization. According to the tutorial, the data was first reduced to 500-dim using PCA beform applying SIMLR. Then, minibatch k-means was used according to its recommended pipeline. For MAGIC, we first imputed the single-cell data using their default parameters. Then we applied PCA to reduce the data to 100 or 256 dimensions, followed by k-means++ clustering. ZIFA is a dimension reduction method that models the dropout characteristics. We used the default dimension to reduce the dimensionality to 2 (default), with and without cell normalization. For PCA and MDS (scikit-learn python package, with default parameters), we reduced the data to both 2-dim and 256-dim and applied k-means++ clustering. Finally, we used the default parameters for SC3.

For pipelines based on k-means, we set k to the actual number of cell labels for comparison. We set the same number of initialization steps (n_init/repeat times $r$) for RPH-kmeans, kmeans++, and k-means (random initialization).

To better benchmark the performance of each algorithm, we performed the algorithm multiple times to obtain a distribution of its performance. Specifically, scDeepCluster and SC3 were run 5 times because there is no intermediate steps in clustering. For methods that involved a dimension reduction step, we generated 5 embeddings with the same parameters, and then applied clustering 10 times to each respective embedding (totals to 50 clustering results). Empty cells in each of the following table indicate that the experiment was not performed either because of insufficient memory or running time was greater than 4 hours. For ease of comparison, we added an asterisk (*) mark to denote the better result of the parameters compared in each benchmarked method (Table S15-21).

## Table S14. Raw performance for compared methods

| Methods | Mouse brain | | PBMC 68k | | Mouse retina | | PBMC 4k | | Mouse bladder | | Mouse ES | | Worm neuron | |
|---|---|---|---|---|---|---|---|---|---|---|---|---|---|---|
| | ARI | NMI | ARI | NMI | ARI | NMI | ARI | NMI | ARI | NMI | ARI | NMI | ARI | NMI |
| **Dim-raw; phenograph** | 0.298 (0.010) | 0.658 (0.003) | 0.505 (0.007) | 0.651 (0.002) | 0.524 (0.000) | 0.635 (0.001) | 0.625 (0.041) | 0.744 (0.008) | 0.453 (0.003) | 0.697 (0.004) | 0.863 (0.000) | 0.898 (0.000) | 0.408 (0.008) | 0.589 (0.015) |
| **Dim-raw; k-means++** | 0.779 (0.097) | 0.847 (0.042) | 0.578 (0.064) | 0.717 (0.032) | 0.546 (0.112) | 0.712 (0.035) | 0.739 (0.054) | 0.784 (0.020) | 0.566 (0.074) | 0.734 (0.022) | 0.770 (0.144) | 0.825 (0.073) | 0.434 (0.056) | 0.620 (0.038) |
| **scDeepCluster** | 0.456 (0.042) | 0.667 (0.030) | 0.562 (0.025) | 0.701 (0.005) | 0.444 (0.016) | 0.793 (0.007) | 0.647 (0.053) | 0.736 (0.014) | 0.519 (0.043) | 0.722 (0.007) | 0.546 (0.008) | 0.678 (0.006) | 0.511 (0.071) | 0.690 (0.037) |
| **DCA** | | | | | 0.552 (0.069) | 0.821 (0.018) | 0.800 (0.012) | 0.800 (0.009) | 0.513 (0.037) | 0.741 (0.013) | 0.946 (0.051) | 0.930 (0.038) | 0.524 (0.049) | 0.719 (0.019) |
| **scVI** | 0.498 (0.023) | 0.718 (0.022) | 0.417 (0.028) | 0.594 (0.019) | 0.388 (0.027) | 0.724 (0.017) | 0.732 (0.044) | 0.752 (0.026) | 0.478 (0.022) | 0.728 (0.011) | 0.671 (0.127) | 0.739 (0.098) | 0.447 (0.021) | 0.626 (0.012) |
| **scScope** | 0.708 (0.022) | 0.819 (0.009) | 0.426 (0.007) | 0.599 (0.012) | 0.311 (0.025) | 0.623 (0.021) | 0.647 (0.017) | 0.729 (0.013) | 0.472 (0.028) | 0.698 (0.009) | 0.785 (0.004) | 0.780 (0.006) | 0.258 (0.017) | 0.444 (0.018) |
| **SIMLR** | 0.276 (0.018) | 0.483 (0.011) | 0.382 (0.029) | 0.522 (0.026) | 0.703 (0.123) | 0.768 (0.026) | 0.609 (0.057) | 0.690 (0.027) | 0.493 (0.042) | 0.694 (0.020) | 0.616 (0.072) | 0.669 (0.058) | 0.281 (0.069) | 0.606 (0.033) |
| **MAGIC** | 0.717 (0.024) | 0.838 (0.009) | 0.463 (0.032) | 0.687 (0.007) | 0.639 (0.021) | 0.796 (0.009) | 0.693 (0.000) | 0.772 (0.001) | 0.606 (0.004) | 0.774 (0.002) | 0.787 (0.001) | 0.838 (0.001) | 0.366 (0.011) | 0.651 (0.012) |
| **SC3** | | | 0.513 (0.017) | 0.607 (0.008) | 0.441 (0.017) | 0.637 (0.011) | 0.737 (0.004) | 0.745 (0.004) | 0.638 (0.041) | 0.750 (0.011) | 0.935 (0.111) | 0.952 (0.080) | 0.297 (0.021) | 0.480 (0.012) |
| **ZIFA** | | | | | | | 0.504 (0.051) | 0.596 (0.028) | 0.363 (0.008) | 0.619 (0.004) | 0.940 (0.001) | 0.916 (0.001) | 0.200 (0.003) | 0.360 (0.002) |
| **MDS** | | | | | | | 0.652 (0.065) | 0.723 (0.017) | 0.483 (0.067) | 0.653 (0.024) | 0.799 (0.049) | 0.835 (0.021) | 0.240 (0.024) | 0.370 (0.015) |
| **AE** | 0.592 (0.065) | 0.785 (0.030) | 0.567 (0.022) | 0.701 (0.011) | 0.428 (0.046) | 0.753 (0.018) | 0.674 (0.071) | 0.740 (0.025) | 0.484 (0.036) | 0.701 (0.011) | 0.551 (0.009) | 0.694 (0.012) | 0.515 (0.065) | 0.661 (0.031) |
| **PCA-2** | 0.447 (0.001) | 0.638 (0.000) | 0.412 (0.004) | 0.583 (0.000) | 0.269 (0.014) | 0.499 (0.002) | 0.729 (0.004) | 0.733 (0.002) | 0.382 (0.013) | 0.600 (0.005) | 0.775 (0.000) | 0.824 (0.000) | 0.184 (0.001) | 0.339 (0.001) |
| **PCA-256** | 0.736 (0.031) | 0.837 (0.009) | 0.599 (0.037) | 0.726 (0.018) | 0.477 (0.052) | 0.78 (0.020) | 0.8 (0.054) | 0.807 (0.022) | 0.541 (0.041) | 0.748 (0.015) | 0.82 (0.072) | 0.853 (0.041) | 0.484 (0.049) | 0.66 (0.024) |
| **AIDE (best); RPH-kmeans (best)** | **0.957 (0.005)** | **0.924 (0.005)** | **0.639 (0.003)** | **0.750 (0.001)** | **0.875 (0.055)** | **0.825 (0.023)** | **0.833 (0.003)** | **0.817 (0.001)** | **0.646 (0.022)** | **0.760 (0.005)** | **0.981 (0.002)** | **0.963 (0.003)** | **0.635 (0.015)** | **0.751 (0.007)** |

Note: Bolded values denote the best performance of each dataset (column). For 'Dim-raw; phenograph' and 'Dim-raw; k-means++', we ran clustering 10 times (5 times for 'Mouse brain') using the pre-processed (cell norm and log transformation) gene expression vector.

## Table S15. Performance of scDeepCluster

| Methods | Mouse brain | | PBMC 68k | | Shekhar | | 10X PBMC | | Mouse bladder | | Mouse ES | | Worm neuron | |
|---|---|---|---|---|---|---|---|---|---|---|---|---|---|---|
| | ARI | NMI | ARI | NMI | ARI | NMI | ARI | NMI | ARI | NMI | ARI | NMI | ARI | NMI |
| **Dim-32 *** | 0.456 (0.042) | 0.667 (0.030) | 0.562 (0.025) | 0.701 (0.005) | 0.444 (0.016) | 0.793 (0.007) | 0.647 (0.053) | 0.736 (0.014) | 0.519 (0.043) | 0.722 (0.007) | 0.546 (0.008) | 0.678 (0.006) | 0.511 (0.071) | 0.690 (0.037) |
| **Dim-256** | 0.513 (0.035) | 0.627 (0.022) | 0.471 (0.044) | 0.645 (0.021) | 0.446 (0.044) | 0.764 (0.017) | 0.686 (0.020) | 0.731 (0.008) | 0.446 (0.016) | 0.668 (0.007) | 0.342 (0.036) | 0.501 (0.024) | 0.422 (0.075) | 0.619 (0.052) |

## Table S16. Performance of DCA

| Methods | Mouse brain | | PBMC 68k | | Mouse retina | | PBMC 4k | | Mouse bladder | | Mouse ES | | Worm neuron | |
|---|---|---|---|---|---|---|---|---|---|---|---|---|---|---|
| | ARI | NMI | ARI | NMI | ARI | NMI | ARI | NMI | ARI | NMI | ARI | NMI | ARI | NMI |
| **Dim-32; hidden** | | | | | | | 0.33 (0.032) | 0.464 (0.0307) | 0.305 (0.022) | 0.577 (0.014) | 0.332 (0.031) | 0.481 (0.036) | 0.096 (0.010) | 0.316 (0.014) |
| **Dim-32; impute** | | | | | 0.499 (0.022) | 0.788 (0.011) | 0.798 (0.010) | 0.8 (0.007) | 0.53 (0.018) | 0.751 (0.006) | 0.868 (0.043) | 0.87 (0.029) | 0.545 (0.032) | 0.723 (0.016) |
| **Dim-32; impute; PCA-256** | | | | | 0.502 (0.017) | 0.79 (0.009) | 0.797 (0.010) | 0.8 (0.008) | 0.52 (0.024) | 0.748 (0.010) | 0.869 (0.043) | 0.871 (0.029) | 0.548 (0.036) | 0.721 (0.018) |
| **Dim-256; hidden** | | | | | | | 0.206 (0.051) | 0.287 (0.066) | 0.284 (0.037) | 0.565 (0.029) | 0.211 (0.011) | 0.289 (0.019) | 0.0349 (0.029) | 0.0991 (0.053) |
| **Dim-256; impute** | | | | | 0.552 (0.064) | 0.825 (0.017) | 0.798 (0.017) | 0.799 (0.009) | 0.517 (0.040) | 0.741 (0.015) | 0.948 (0.050) | 0.932 (0.037) | 0.52 (0.054) | 0.716 (0.022) |
| **Dim-256; impute; PCA-256 *** | | | | | 0.552 (0.069) | 0.821 (0.018) | 0.8 (0.012) | 0.8 (0.009) | 0.513 (0.038) | 0.741 (0.013) | 0.946 (0.051) | 0.93 (0.038) | 0.524 (0.049) | 0.719 (0.019) |

Note: 'hidden' denotes that we applied clustering directly on hidden vectors produced by DCA; 'impute' denotes that we applied clustering on imputed gene expression vectors; 'impute; PCA' means that we first applied PCA to reduce the dimensions of the imputed gene expression vectors before clustering. We did not benchmark DCA with 'hidden' on the Mouse retina dataset because the performance was poor on four small datasets (PBMC 4k, Mouse bladder, Mouse ES, and Worm neuron).

## Table S17. Performance of scVI

| Methods | Mouse brain | | PBMC 68k | | Mouse retina | | PBMC 4k | | Mouse bladder | | Mouse ES | | Worm neuron | |
|---|---|---|---|---|---|---|---|---|---|---|---|---|---|---|
| | ARI | NMI | ARI | NMI | ARI | NMI | ARI | NMI | ARI | NMI | ARI | NMI | ARI | NMI |
| **Dim-10; k-means++ *** | 0.498 (0.023) | 0.718 (0.022) | 0.417 (0.028) | 0.594 (0.019) | 0.388 (0.027) | 0.724 (0.017) | 0.732 (0.044) | 0.752 (0.026) | 0.478 (0.022) | 0.728 (0.011) | 0.671 (0.127) | 0.739 (0.098) | 0.447 (0.021) | 0.626 (0.012) |
| **Dim-256; k-means++** | 0.507 (0.030) | 0.723 (0.025) | 0.470 (0.022) | 0.633 (0.010) | 0.319 (0.021) | 0.655 (0.024) | 0.592 (0.035) | 0.702 (0.018) | 0.440 (0.027) | 0.691 (0.018) | 0.470 (0.030) | 0.585 (0.026) | 0.432 (0.025) | 0.599 (0.019) |

## Table S18. Performance of scScope

| Methods | Mouse brain | | PBMC 68k | | Mouse retina | | PBMC 4k | | Mouse bladder | | Mouse ES | | Worm neuron | |
|---|---|---|---|---|---|---|---|---|---|---|---|---|---|---|
| | ARI | NMI | ARI | NMI | ARI | NMI | ARI | NMI | ARI | NMI | ARI | NMI | ARI | NMI |
| **Dim-50; phenograph** | 0.215 (0.023) | 0.572 (0.006) | 0.189 (0.022) | 0.365 (0.015) | 0.276 (0.024) | 0.447 (0.011) | 0.404 (0.028) | 0.601 (0.012) | 0.438 (0.014) | 0.658 (0.015) | 0.172 (0.015) | 0.375 (0.010) | 0.072 (0.008) | 0.225 (0.012) |
| **Dim-50; k-means++** | 0.038 (0.027) | 0.115 (0.069) | 0.165 (0.018) | 0.297 (0.026) | 0.164 (0.015) | 0.309 (0.011) | 0.437 (0.012) | 0.555 (0.014) | 0.398 (0.027) | 0.640 (0.020) | 0.423 (0.004) | 0.504 (0.007) | 0.071 (0.006) | 0.134 (0.007) |
| **Dim-256; phenograph** | 0.228 (0.016) | 0.583 (0.005) | 0.171 (0.014) | 0.327 (0.012) | 0.262 (0.029) | 0.420 (0.012) | 0.451 (0.027) | 0.591 (0.011) | 0.456 (0.038) | 0.622 (0.014) | 0.457 (0.098) | 0.597 (0.058) | 0.046 (0.008) | 0.191 (0.015) |
| **Dim-256; k-means++** | 0.002 (0.002) | 0.044 (0.004) | 0.141 (0.018) | 0.256 (0.022) | 0.173 (0.015) | 0.304 (0.012) | 0.402 (0.025) | 0.514 (0.022) | 0.351 (0.020) | 0.587 (0.016) | 0.453 (0.018) | 0.573 (0.040) | 0.047 (0.010) | 0.117 (0.012) |
| **Dim-50; gene-1000; phenograph** | 0.194 (0.008) | 0.658 (0.003) | 0.222 (0.012) | 0.444 (0.006) | 0.662 (0.076) | 0.750 (0.012) | 0.524 (0.050) | 0.657 (0.013) | 0.555 (0.017) | 0.731 (0.008) | 0.656 (0.095) | 0.733 (0.027) | 0.346 (0.010) | 0.584 (0.012) |
| **Dim-50; gene-1000; k-means++ *** | 0.708 (0.022) | 0.819 (0.009) | 0.426 (0.007) | 0.599 (0.012) | 0.311 (0.025) | 0.623 (0.021) | 0.647 (0.017) | 0.729 (0.013) | 0.472 (0.028) | 0.698 (0.009) | 0.785 (0.004) | 0.780 (0.006) | 0.258 (0.017) | 0.444 (0.018) |
| **Dim-256; gene-1000; phenograph** | 0.213 (0.013) | 0.665 (0.006) | 0.196 (0.013) | 0.433 (0.007) | 0.562 (0.048) | 0.597 (0.017) | 0.591 (0.067) | 0.686 (0.020) | 0.501 (0.007) | 0.678 (0.004) | 0.278 (0.136) | 0.535 (0.108) | 0.304 (0.015) | 0.529 (0.008) |
| **Dim-256; gene-1000; k-means++** | 0.643 (0.056) | 0.740 (0.024) | 0.419 (0.005) | 0.605 (0.010) | 0.241 (0.017) | 0.515 (0.006) | 0.627 (0.018) | 0.709 (0.014) | 0.415 (0.024) | 0.658 (0.008) | 0.668 (0.144) | 0.660 (0.105) | 0.236 (0.017) | 0.427 (0.019) |

Note: For the Mouse brain dataset, 'phenograph' refers to the 'phenograph+kmeans' method proposed in the paper of scScope (M = 200, K = 100). For the other datasets, 'phenograph' refers to the original Phenograph clustering method with default parameters.

## Table S19. Performance of SIMLR

| Methods | Mouse brain | | PBMC 68k | | Mouse retina | | PBMC 4k | | Mouse bladder | | Mouse ES | | Worm neuron | |
|---|---|---|---|---|---|---|---|---|---|---|---|---|---|---|
| | ARI | NMI | ARI | NMI | ARI | NMI | ARI | NMI | ARI | NMI | ARI | NMI | ARI | NMI |
| **Default** | 0.245 (0.058) | 0.425 (0.027) | 0.232 (0.021) | 0.404 (0.032) | 0.387 (0.060) | 0.582 (0.029) | 0.528 (0.070) | 0.660 (0.031) | 0.552 (0.056) | 0.728 (0.028) | 0.462 (0.019) | 0.581 (0.023) | 0.256 (0.069) | 0.592 (0.038) |
| **Default; cell norm *** | 0.276 (0.018) | 0.483 (0.011) | 0.382 (0.029) | 0.522 (0.026) | 0.703 (0.123) | 0.768 (0.026) | 0.609 (0.057) | 0.690 (0.027) | 0.493 (0.042) | 0.694 (0.020) | 0.616 (0.072) | 0.669 (0.058) | 0.281 (0.069) | 0.606 (0.033) |

## Table S20. Performance of MAGIC

| Methods | Mouse brain | | PBMC 68k | | Mouse retina | | PBMC 4k | | Mouse bladder | | Mouse ES | | Worm neuron | |
|---|---|---|---|---|---|---|---|---|---|---|---|---|---|---|
| | ARI | NMI | ARI | NMI | ARI | NMI | ARI | NMI | ARI | NMI | ARI | NMI | ARI | NMI |
| **Impute; PCA-100; k-means++ *** | 0.717 (0.024) | 0.838 (0.009) | 0.463 (0.032) | 0.687 (0.007) | 0.639 (0.021) | 0.796 (0.009) | 0.693 (0.000) | 0.772 (0.001) | 0.606 (0.004) | 0.774 (0.002) | 0.787 (0.001) | 0.838 (0.001) | 0.366 (0.011) | 0.651 (0.012) |
| **Impute; PCA-256; k-means++** | 0.739 (0.030) | 0.843 (0.010) | 0.387 (0.001) | 0.644 (0.000) | 0.929 (0.004) | 0.851 (0.005) | 0.685 (0.003) | 0.759 (0.001) | 0.533 (0.008) | 0.737 (0.002) | 0.790 (0.001) | 0.835 (0.001) | 0.262 (0.064) | 0.534 (0.010) |

## Table S21. Performance of ZIFA

| Methods | Mouse brain | | PBMC 68k | | Mouse retina | | PBMC 4k | | Mouse bladder | | Mouse ES | | Worm neuron | |
|---|---|---|---|---|---|---|---|---|---|---|---|---|---|---|
| | ARI | NMI | ARI | NMI | ARI | NMI | ARI | NMI | ARI | NMI | ARI | NMI | ARI | NMI |
| **Dim-2; k-means++** | | | | | | | 0.230 (0.001) | 0.380 (0.001) | 0.197 (0.004) | 0.445 (0.005) | 0.772 (0.002) | 0.746 (0.001) | 0.108 (0.002) | 0.216 (0.001) |
| **Dim-2; cell norm; k-means++ *** | | | | | | | 0.504 (0.051) | 0.596 (0.028) | 0.363 (0.008) | 0.619 (0.004) | 0.940 (0.001) | 0.916 (0.001) | 0.200 (0.003) | 0.360 (0.002) |

# Supplementary Note IV

## Comparison of RPH-kmeans against k-means algorithms

Here, we compared the clustering performance of RPH-kmeans against k-means++ and k-means (random initialization) on benchmarked datasets. We first performed either PCA (with 256 dimensions) or AIDE embedding on the 7 datasets (5 different embeddings were generated for each dataset), then applied the clustering algorithm 10 times. We set k to the actual number of cell types and the number of initializations (n_init/repeat times $r$) to 10 for RPH-kmeans, kmeans++, and k-means (random initialization). The results in the following table (Table S22) show the average evaluation based on 50 clustering results with standard deviation in brackets. We observe that RPH-kmeans outperforms the other k-means algorithms in most datasets, especially in 'Mouse retina' and 'Mouse bladder'. In both of these datasets, the distribution of cell types is highly imbalanced with many small clusters, suggesting that RPH-kmeans favors the detection of small cell clusters. This leads to our rare cell type simulation described in Note V, where we explored the performance of RPH-kmeans in detecting rare cell types.

**Table S22. Raw performance for different k-means algorithms**

| Methods | Mouse brain | | PBMC 68k | | Mouse retina | | PBMC 4k | | Mouse bladder | | Mouse ES | | Worm neuron | |
|---|---|---|---|---|---|---|---|---|---|---|---|---|---|---|
| | ARI | NMI | ARI | NMI | ARI | NMI | ARI | NMI | ARI | NMI | ARI | NMI | ARI | NMI |
| **PCA-256; k-means (random)** | 0.696 (0.093) | 0.824 (0.036) | 0.58 (0.053) | 0.716 (0.027) | 0.442 (0.048) | 0.761 (0.019) | 0.793 (0.054) | 0.803 (0.022) | 0.588 (0.049) | 0.755 (0.018) | 0.785 (0.025) | 0.833 (0.016) | 0.489 (0.052) | 0.661 (0.025) |
| **PCA-256; k-means++** | 0.736 (0.031) | 0.837 (0.009) | 0.599 (0.037) | 0.726 (0.018) | 0.477 (0.052) | 0.78 (0.020) | 0.8 (0.054) | 0.807 (0.022) | 0.541 (0.041) | 0.748 (0.015) | 0.82 (0.072) | 0.853 (0.041) | 0.484 (0.049) | 0.66 (0.024) |
| **PCA-256; RPH-kmeans (default)** | **0.743 (0.000)** | **0.838 (0.000)** | **0.62 (0.042)** | **0.733 (0.025)** | **0.61 (0.121)** | 0.764 (0.034) | **0.826 (0.032)** | **0.817 (0.012)** | **0.644 (0.038)** | **0.778 (0.011)** | 0.782 (0.001) | 0.832 (0.001) | **0.49 (0.050)** | 0.658 (0.027) |
| **PCA-256; RPH-kmeans (best)** | **0.743 (0.000)** | **0.838 (0.000)** | **0.653 (0.017)** | **0.752 (0.008)** | **0.631 (0.059)** | **0.802 (0.020)** | **0.836 (0.002)** | **0.819 (0.001)** | **0.651 (0.033)** | **0.771 (0.010)** | 0.782 (0.001) | 0.833 (0.001) | **0.534 (0.052)** | **0.686 (0.026)** |
| **AIDE (best); k-means (random)** | 0.784 (0.141) | 0.864 (0.049) | 0.572 (0.037) | 0.714 (0.018) | 0.514 (0.068) | 0.764 (0.017) | 0.805 (0.040) | 0.805 (0.017) | 0.521 (0.057) | 0.719 (0.014) | 0.981 (0.002) | 0.963 (0.003) | 0.591 (0.053) | 0.735 (0.016) |
| **AIDE (best); k-means++** | 0.957 (0.006) | 0.923 (0.006) | 0.635 (0.011) | 0.748 (0.006) | 0.716 (0.135) | 0.808 (0.024) | 0.824 (0.038) | 0.813 (0.016) | 0.630 (0.030) | 0.757 (0.006) | 0.981 (0.002) | 0.963 (0.003) | 0.625 (0.040) | 0.747 (0.014) |
| **AIDE (best); RPH-kmeans (default)** | **0.957 (0.005)** | **0.924 (0.005)** | **0.639 (0.004)** | **0.75 (0.003)** | **0.875 (0.055)** | **0.825 (0.023)** | 0.821 (0.038) | 0.812 (0.015) | 0.554 (0.027) | 0.736 (0.011) | **0.981 (0.002)** | **0.963 (0.003)** | **0.633 (0.029)** | **0.748 (0.012)** |
| **AIDE (best); RPH-kmeans (best)** | **0.957 (0.005)** | **0.924 (0.005)** | **0.639 (0.003)** | **0.750 (0.001)** | **0.875 (0.055)** | **0.825 (0.023)** | **0.833 (0.003)** | **0.817 (0.001)** | **0.646 (0.022)** | **0.760 (0.005)** | **0.981 (0.002)** | **0.963 (0.003)** | **0.635 (0.015)** | **0.751 (0.007)** |

Note: "best" and "default" denote the tuned and default parameters respectively. Bolded values denote that the performance is equal to or better than the better result of k-means (random) and k-means++.

# Supplementary Note V

## Simulation experiments on detection of rare cell types

Detecting rare cell type subpopulations is one of the important applications in single-cell analysis. For example, within a specific tumor micro-environment, the composition of cell types may be highly imbalanced in numbers. In general, existing clustering methods often detect the larger clusters well but miss out the smaller groups. Here, we present results for simulation experiments as mentioned in the results section. We increased the imbalance of cell types within datasets by retaining the largest two cell groups, then sampled 50 or 500 cells (50 if the size of the dataset was less than 10,000 cells) for the remaining groups. If the cell group contained a smaller number of cells than the sampled number, we simply retain all cells of that particular group (Table S23). We show that RPH-kmeans is tailored for detection of rare cell types by comparing its clustering performance on seven simulated datasets (Table S24).

**Table S23. Group sizes of original/imbalanced datasets**

| Datasets | Cell | No. of cell types | Group size |
|---|---|---|---|
| Mouse brain | 160,796 | 7 | [74539, 31073, 22491, 12196, 11217, 7454, 1826] |
| Mouse brain (I) | 108,112 | 7 | [74539, 31073, 500, 500, 500, 500, 500] |
| PBMC 68k | 68,579 | 10 | [21429, 11652, 8570, 7656, 6398, 4492, 3941, 3923, 342, 176] |
| PBMC 68k (I) | 36,599 | 10 | [21429, 11652, 500, 500, 500, 500, 500, 500, 342, 176] |
| Mouse retina | 27,499 | 19 | [10888, 2945, 2237, 1759, 1702, 1372, 1091, 817, 792, 669, 558, 553, 535, 479, 398, 313, 252, 91, 48] |
| Mouse retina (I) | 20,914 | 19 | [10888, 2945, 500, 500, 500, 500, 500, 500, 500, 500, 500, 500, 500, 479, 398, 313, 252, 91, 48] |
| PBMC 4k | 4,271 | 8 | [1292, 702, 606, 459, 450, 332, 295, 135] |
| PBMC 4k (I) | 2,294 | 8 | [1292, 702, 50, 50, 50, 50, 50, 50] |
| Mouse bladder | 2,746 | 16 | [717, 357, 344, 316, 236, 224, 131, 80, 75, 64, 44, 41, 38, 36, 30, 13] |
| Mouse bladder (I) | 1,676 | 16 | [717, 357, 50, 50, 50, 50, 50, 50, 50, 50, 44, 41, 38, 36, 30, 13] |
| Mouse ES | 2,717 | 4 | [933, 798, 683, 303] |
| Mouse ES (I) | 1,831 | 4 | [933, 798, 50, 50] |
| Worm neuron | 4,186 | 10 | [1015, 842, 443, 400, 334, 314, 305, 239, 224, 70] |
| Worm neuron (I) | 2,257 | 10 | [1015, 842, 50, 50, 50, 50, 50, 50, 50, 50] |

Note: '(I)' denotes the simulated imbalanced dataset.

**Table S24. Raw performance of different k-means on simulated imbalanced datasets**

| Methods | Mouse brain (I) | | PBMC 68k (I) | | Mouse retina (I) | | PBMC 4k (I) | | Mouse bladder (I) | | Mouse ES (I) | | Worm neuron (I) | |
|---|---|---|---|---|---|---|---|---|---|---|---|---|---|---|
| | ARI | NMI | ARI | NMI | ARI | NMI | ARI | NMI | ARI | NMI | ARI | NMI | ARI | NMI |
| **PCA-256; k-means (random; n_init = 1)** | 0.288 (0.043) | 0.532 (0.026) | 0.258 (0.039) | 0.473 (0.021) | 0.233 (0.046) | 0.624 (0.032) | 0.679 (0.160) | 0.723 (0.067) | 0.456 (0.089) | 0.674 (0.034) | 0.668 (0.258) | 0.676 (0.185) | 0.291 (0.066) | 0.429 (0.040) |
| **PCA-256; k-means++ (n_init = 1)** | 0.274 (0.034) | 0.523 (0.025) | 0.254 (0.033) | 0.472 (0.018) | 0.284 (0.059) | 0.673 (0.034) | 0.632 (0.160) | 0.707 (0.065) | 0.434 (0.083) | 0.691 (0.032) | 0.683 (0.258) | 0.698 (0.174) | 0.249 (0.051) | 0.431 (0.036) |
| **PCA-256; RPH-kmeans (default; n_init = 1)** | **0.340 (0.056)** | **0.564 (0.029)** | **0.544 (0.100)** | **0.590 (0.045)** | **0.562 (0.157)** | **0.695 (0.049)** | **0.838 (0.103)** | **0.793 (0.049)** | **0.557 (0.064)** | **0.731 (0.023)** | **0.799 (0.150)** | **0.786 (0.100)** | **0.323 (0.065)** | **0.456 (0.044)** |
| **PCA-256; k-means (random; n_init = 10)** | 0.270 (0.024) | 0.523 (0.023) | 0.294 (0.048) | 0.494 (0.018) | 0.289 (0.049) | 0.676 (0.024) | 0.601 (0.096) | 0.706 (0.034) | 0.478 (0.078) | 0.707 (0.029) | 0.831 (0.038) | 0.818 (0.029) | 0.247 (0.041) | 0.448 (0.033) |
| **PCA-256; k-means++ (n_init = 10)** | 0.274 (0.021) | 0.526 (0.019) | 0.296 (0.059) | 0.495 (0.021) | 0.312 (0.046) | 0.697 (0.022) | 0.574 (0.107) | 0.699 (0.037) | 0.428 (0.072) | 0.705 (0.028) | 0.835 (0.031) | 0.821 (0.021) | 0.236 (0.029) | 0.450 (0.025) |
| **PCA-256; RPH-kmeans (default; n_init = 10)** | **0.293 (0.019)** | **0.536 (0.013)** | **0.467 (0.084)** | **0.557 (0.031)** | **0.491 (0.086)** | **0.710 (0.030)** | **0.744 (0.126)** | **0.759 (0.042)** | **0.540 (0.063)** | **0.742 (0.025)** | **0.839 (0.001)** | **0.825 (0.002)** | **0.269 (0.033)** | **0.452 (0.033)** |
| **AIDE (default); k-means (random; n_init = 1)** | 0.313 (0.064) | 0.546 (0.021) | 0.287 (0.023) | 0.488 (0.012) | 0.194 (0.065) | 0.559 (0.027) | 0.574 (0.107) | 0.705 (0.033) | 0.276 (0.056) | 0.630 (0.032) | 0.785 (0.126) | 0.807 (0.087) | 0.202 (0.026) | 0.436 (0.025) |
| **AIDE (default); k-means++ (n_init = 1)** | 0.307 (0.020) | 0.554 (0.019) | 0.350 (0.077) | 0.514 (0.032) | 0.356 (0.087) | 0.609 (0.038) | 0.628 (0.134) | 0.729 (0.042) | 0.561 (0.070) | **0.752 (0.018)** | **0.867 (0.001)** | **0.860 (0.001)** | 0.219 (0.029) | 0.447 (0.025) |
| **AIDE (default); RPH-kmeans (default; n_init = 1)** | **0.359 (0.048)** | **0.586 (0.027)** | **0.582 (0.056)** | **0.598 (0.031)** | **0.880 (0.054)** | **0.728 (0.025)** | **0.810 (0.109)** | **0.784 (0.036)** | **0.627 (0.087)** | 0.749 (0.022) | 0.854 (0.093) | 0.851 (0.062) | **0.225 (0.028)** | **0.459 (0.023)** |
| **AIDE (default); k-means (random; n_init = 10)** | 0.302 (0.017) | 0.546 (0.011) | 0.293 (0.009) | 0.494 (0.009) | 0.243 (0.049) | 0.578 (0.027) | 0.582 (0.121) | 0.714 (0.041) | 0.349 (0.061) | 0.679 (0.024) | 0.866 (0.007) | 0.858 (0.013) | 0.210 (0.021) | 0.451 (0.018) |
| **AIDE (default); k-means++ (n_init = 10)** | 0.300 (0.011) | 0.544 (0.008) | 0.389 (0.093) | 0.539 (0.032) | 0.415 (0.079) | 0.627 (0.039) | 0.748 (0.159) | 0.765 (0.052) | 0.569 (0.039) | 0.759 (0.012) | 0.867 (0.001) | 0.860 (0.001) | 0.215 (0.024) | 0.453 (0.021) |
| **AIDE (default); RPH-kmeans (default; n_init = 10)** | **0.318 (0.021)** | **0.560 (0.021)** | **0.530 (0.074)** | **0.582 (0.030)** | **0.810 (0.154)** | **0.718 (0.031)** | **0.794 (0.129)** | **0.779 (0.043)** | **0.690 (0.044)** | **0.766 (0.015)** | **0.867 (0.001)** | **0.860 (0.001)** | **0.222 (0.023)** | **0.466 (0.018)** |

Note: For Mouse bladder (I) and Mouse ES (I), the maximum number of skeleton points of RPH-kmeans is set to 1,500 as the size of the datasets are less than 2000 (default maximum number of skeleton points). The bolded values represent the best performance out of k-means (random initialization), k-means++, and RPH-kmeans for each embedding (PCA and AIDE with different parameters). n_init of RPH-kmeans refers to the repeat times $r$ in algorithm 1.

# Supplementary Note VI

## Gene Selection

Although some studies require a gene selection process prior to clustering analysis, we believe that minimal pre-processing preserves the most information about each single-cell. In the following table (Table S25), we compared the performance of full gene expression against selected gene expression.

We followed a conventional gene expression selection process similar to scScope. First, the gene expression is pre-processed by filtering cells and genes with a minimum count of 1. Then we applied cell normalization followed by log transformation. The top 1,000 highly variable genes were selected based on the dispersion ratio (variance / mean).

Bold values represent the better result of using full gene expression versus selected gene expression. Using PCA + k-means++ clustering as a simple baseline, we observe that full gene expression performs better in most datasets (6 out of 7). With AIDE embedding, better clustering results were achieved using the full gene expression in almost all cases.

**Table S25. Full Gene Expression vs. Gene Selection**

| Methods | Mouse brain | | PBMC 68k | | Mouse retina | | PBMC 4k | | Mouse bladder | | Mouse ES | | Worm neuron | |
|---|---|---|---|---|---|---|---|---|---|---|---|---|---|---|
| | ARI | NMI | ARI | NMI | ARI | NMI | ARI | NMI | ARI | NMI | ARI | NMI | ARI | NMI |
| **PCA-256; k-means++** | 0.736 (0.031) | 0.837 (0.009) | **0.599 (0.037)** | **0.726 (0.018)** | **0.477 (0.052)** | **0.780 (0.020)** | **0.800 (0.054)** | **0.807 (0.022)** | **0.541 (0.041)** | **0.748 (0.015)** | **0.820 (0.072)** | **0.853 (0.041)** | **0.484 (0.049)** | **0.660 (0.024)** |
| **Gene-1000; PCA-256; k-means++** | **0.935 (0.000)** | **0.883 (0.000)** | 0.427 (0.016) | 0.637 (0.021) | 0.449 (0.073) | 0.764 (0.018) | 0.647 (0.023) | 0.726 (0.011) | 0.477 (0.030) | 0.704 (0.011) | 0.808 (0.001) | 0.804 (0.001) | 0.352 (0.049) | 0.607 (0.025) |
| **PCA-256; RPH-kmeans** | 0.741 (0.011) | 0.838 (0.000) | **0.620 (0.042)** | **0.733 (0.025)** | 0.610 (0.121) | 0.764 (0.034) | **0.826 (0.032)** | **0.817 (0.012)** | **0.644 (0.038)** | **0.778 (0.011)** | 0.782 (0.001) | **0.832 (0.001)** | **0.490 (0.050)** | **0.658 (0.027)** |
| **Gene-1000; PCA-256; RPH-kmeans** | **0.935 (0.000)** | **0.883 (0.000)** | 0.440 (0.002) | 0.656 (0.004) | **0.731 (0.137)** | **0.778 (0.032)** | 0.637 (0.007) | 0.721 (0.006) | 0.534 (0.032) | 0.722 (0.011) | **0.809 (0.001)** | 0.805 (0.001) | 0.320 (0.049) | 0.587 (0.029) |
| **AIDE-256; k-means++** | **0.957 (0.006)** | **0.923 (0.006)** | **0.635 (0.011)** | **0.748 (0.006)** | **0.716 (0.135)** | **0.808 (0.024)** | **0.824 (0.038)** | **0.813 (0.016)** | **0.630 (0.030)** | **0.757 (0.006)** | **0.981 (0.002)** | **0.963 (0.003)** | **0.625 (0.040)** | **0.747 (0.014)** |
| **Gene-1000; AIDE-256; k-means++** | 0.952 (0.001) | 0.906 (0.001) | 0.444 (0.001) | 0.661 (0.009) | 0.705 (0.142) | 0.801 (0.025) | 0.666 (0.023) | 0.740 (0.012) | 0.572 (0.044) | 0.742 (0.007) | 0.810 (0.001) | 0.821 (0.001) | 0.560 (0.032) | 0.722 (0.015) |
| **AIDE-256; RPH-kmeans** | **0.957 (0.005)** | **0.924 (0.005)** | **0.639 (0.003)** | **0.750 (0.001)** | 0.875 (0.055) | **0.825 (0.023)** | **0.833 (0.003)** | **0.817 (0.001)** | **0.646 (0.022)** | **0.760 (0.005)** | **0.981 (0.002)** | **0.963 (0.003)** | **0.635 (0.015)** | **0.751 (0.007)** |
| **Gene-1000; AIDE-256; RPH-kmeans** | 0.952 (0.001) | 0.906 (0.001) | 0.445 (0.001) | 0.670 (0.001) | **0.885 (0.030)** | 0.804 (0.029) | 0.676 (0.045) | 0.739 (0.013) | 0.596 (0.037) | 0.749 (0.006) | 0.810 (0.001) | 0.821 (0.001) | 0.534 (0.039) | 0.710 (0.010) |

Note: For PCA components, we used the default parameters for RPH-kmeans. AIDE parameters are equivalent to Table S3 for each dataset. We used the same parameters for RPH-kmeans as shown in Table S5. n_init = 10 for both k-means++ and RPH-kmeans.

# Supplementary Note VII

## PCA/AIDE + Other Clustering

In order to show that our AIDE embedding is a good new representation of single-cells, we added experiments to apply conventional clustering methods to AIDE embedding.

Focusing on the PCA components, we can observe that RPH-kmeans consistently achieves a relatively good clustering performance and outperforms the other clustering methods in most datasets.

Comparing the performance of clustering methods applied to PCA and AIDE, we believe that AIDE embedding better captures the cell type composition. In particular, DBSCAN and spectral clustering both performed quite poorly on PCA components. However, the performance significantly increased when applied to AIDE embeddings (especially in Mouse ES, Mouse retina, Mouse bladder, and Worm neuron datasets).

**Table S26. PCA/AIDE + Other Clustering**

| Methods | Mouse brain | | PBMC 68k | | Mouse retina | | PBMC 4k | | Mouse bladder | | Mouse ES | | Worm neuron | |
|---|---|---|---|---|---|---|---|---|---|---|---|---|---|---|
| | ARI | NMI | ARI | NMI | ARI | NMI | ARI | NMI | ARI | NMI | ARI | NMI | ARI | NMI |
| **PCA-256; k-means++** | 0.736 (0.031) | 0.837 (0.009) | 0.599 (0.037) | 0.726 (0.018) | 0.477 (0.052) | 0.780 (0.020) | 0.800 (0.054) | 0.807 (0.022) | 0.541 (0.041) | 0.748 (0.015) | 0.820 (0.072) | 0.853 (0.041) | 0.484 (0.049) | 0.660 (0.024) |
| **PCA-256; RPH-kmeans** | 0.743 (0.000) | 0.838 (0.000) | 0.653 (0.017) | 0.752 (0.008) | 0.631 (0.059) | 0.802 (0.020) | 0.836 (0.002) | 0.819 (0.001) | 0.651 (0.033) | 0.771 (0.010) | 0.782 (0.001) | 0.833 (0.001) | 0.534 (0.052) | 0.686 (0.026) |
| **PCA-256; Phenograph** | 0.150 (0.005) | 0.567 (0.003) | 0.569 (0.006) | 0.701 (0.003) | 0.673 (0.001) | 0.844 (0.001) | 0.785 (0.004) | 0.789 (0.003) | 0.645 (0.007) | 0.764 (0.005) | 0.828 (0.002) | 0.858 (0.002) | 0.366 (0.006) | 0.672 (0.003) |
| **PCA-256; Hierarchical** | | | 0.550 (0.005) | 0.672 (0.002) | 0.657 (0.014) | 0.821 (0.008) | 0.752 (0.009) | 0.764 (0.003) | 0.558 (0.021) | 0.746 (0.003) | 0.776 (0.019) | 0.826 (0.020) | 0.457 (0.053) | 0.631 (0.023) |
| **PCA-256; Gaussian Mix.** | 0.543 (0.035) | 0.779 (0.020) | 0.353 (0.022) | 0.546 (0.016) | 0.463 (0.080) | 0.757 (0.034) | 0.742 (0.070) | 0.785 (0.031) | 0.486 (0.049) | 0.724 (0.017) | 0.732 (0.091) | 0.810 (0.046) | 0.468 (0.067) | 0.650 (0.036) |
| **PCA-256; Spectral** | | | | | | | 0.000 (0.000) | 0.003 (0.001) | 0.001 (0.000) | 0.011 (0.001) | 0.000 (0.001) | 0.003 (0.002) | 0.032 (0.021) | 0.180 (0.072) |
| **PCA-256; DBSCAN** | | | | | | | 0.099 (0.029) | 0.200 (0.078) | 0.105 (0.001) | 0.286 (0.002) | 0.412 (0.002) | 0.480 (0.001) | 0.011 (0.000) | 0.088 (0.001) |
| **AIDE-256; k-means++** | 0.957 (0.006) | 0.923 (0.006) | 0.635 (0.011) | 0.748 (0.006) | 0.716 (0.135) | 0.808 (0.024) | 0.824 (0.038) | 0.813 (0.016) | 0.630 (0.030) | 0.757 (0.006) | 0.981 (0.002) | 0.963 (0.003) | 0.625 (0.040) | 0.747 (0.014) |
| **AIDE-256; RPH-kmeans** | 0.957 (0.005) | 0.924 (0.005) | 0.639 (0.003) | 0.750 (0.001) | 0.875 (0.055) | 0.825 (0.023) | 0.833 (0.003) | 0.817 (0.001) | 0.646 (0.022) | 0.760 (0.005) | 0.981 (0.002) | 0.963 (0.003) | 0.635 (0.015) | 0.751 (0.007) |
| **AIDE-256; Phenograph** | 0.289 (0.011) | 0.657 (0.006) | 0.563 (0.046) | 0.697 (0.014) | 0.763 (0.127) | 0.838 (0.019) | 0.739 (0.029) | 0.778 (0.009) | 0.646 (0.050) | 0.759 (0.008) | 0.825 (0.011) | 0.857 (0.010) | 0.457 (0.011) | 0.749 (0.006) |
| **AIDE-256; Hierarchical** | | | 0.555 (0.023) | 0.705 (0.010) | 0.853 (0.100) | 0.837 (0.015) | 0.754 (0.014) | 0.767 (0.016) | 0.673 (0.065) | 0.763 (0.013) | 0.980 (0.002) | 0.965 (0.002) | 0.616 (0.075) | 0.754 (0.024) |
| **AIDE-256; Gaussian Mix.** | 0.788 (0.065) | 0.814 (0.030) | 0.479 (0.029) | 0.632 (0.017) | 0.722 (0.097) | 0.747 (0.037) | 0.707 (0.067) | 0.769 (0.029) | 0.546 (0.045) | 0.713 (0.013) | 0.811 (0.047) | 0.856 (0.029) | 0.651 (0.026) | 0.755 (0.009) |
| **AIDE-256; Spectral** | | | | | | | 0.000 (0.000) | 0.003 (0.001) | 0.133 (0.027) | 0.448 (0.035) | 0.810 (0.108) | 0.861 (0.064) | 0.526 (0.040) | 0.731 (0.005) |
| **AIDE-256; DBSCAN** | | | | | | | 0.269 (0.006) | 0.385 (0.007) | 0.532 (0.003) | 0.701 (0.005) | 0.769 (0.151) | 0.816 (0.109) | 0.503 (0.037) | 0.686 (0.019) |

Note:
For each dataset, we performed 5 embeddings and applied clustering 10 times to each embedding with a few exceptions (if it took too long or algorithm exceeds the memory threshold).
Hierarchical Clustering (Linkage = Ward): PBMC 68k - applied clustering once to 5 embeddings.
Spectral Clustering: Mouse retina - applied clustering 3 times to 5 embeddings.
Empty cells: Memory error or the algorithm took too long.
Default parameters were used for most methods except for DBSCAN, where we tuned the parameter 'eps' and 'min samples' to ensure better clustering performance. We used the default parameters implemented in the python package sklearn for conventional clustering methods. n_init = 10 for k-means++.
AIDE parameters are equivalent to Table S3 for each dataset and we used the same parameters for RPH-kmeans as shown in Table S5.
We used the same parameters as shown in Table S6 for RPH-kmeans applied to PCA components.
